# Supplementary material for: Predictive accuracy of risk prediction models for recurrence, metastasis and survival for early-stage cutaneous melanoma: a systematic review
Source: BMJ Open. 2023 Sep 28;13(9):e073306. doi: 10.1136/bmjopen-2023-073306 (PMC10546114; doi:10.1136/bmjopen-2023-073306)
Supplement: Supplementary data [file bmjopen-2023-073306supp001.pdf]

Supplementary Materials

Supplemental Table 1: Medline Search Strategy

Originally searched September 2018, update searches in 16th July 2019, and 30th September 2021

Database(s): Ovid MEDLINE(R) and In-Process & Other Non-Indexed Citations 1946 to July 15, 2019

#	Searches	Results
1	(Validat* or Predict* or Rule* or (Predict* and (Outcome* or Risk* or Model*)) or ((History or Variable* or Criteria or Scor* or Characteristic* or Finding* or Factor*) and (Predict* or Model* or Decision* or Identif* or Prognos*))).mp. or (Decision*.mp. and ((Model* or Clinical*).mp. or Logistic Models/)) or (Prognostic and (History or Variable* or Criteria or Scor* or Characteristic* or Finding* or Factor* or Model*)).ti,ab. [mp=title, abstract, original title, name of substance word, subject heading word, floating sub-heading word, keyword heading word, organism supplementary concept word, protocol supplementary concept word, rare disease supplementary concept word, unique identifier, synonyms]	4600871
2	Stratification.mp. or ROC Curve/ or Discrimination.mp. or Discriminate.mp. or c-statistic.mp. or c statistic.mp. or Area under the curve.mp. or AUC.mp. or Calibration.mp. or Indices.mp. or Algorithm.mp. or Multivariable.mp. or Prognosis.mp. [mp=title, abstract, original title, name of substance word, subject heading word, floating sub-heading word, keyword heading word, organism supplementary concept word, protocol supplementary concept word, rare disease supplementary concept word, unique identifier, synonyms]	1450061
3	1 or 2	5285509
4	*melanoma/	65466
5	*skin neoplasms/	99710
6	melanoma.kw.	7937
7	(malignant adj3 melanoma*).ti,ab.	27244
8	(tumo* adj5 (mole or melanoma)).ti,ab.	12160
9	4 or 5 or 6 or 7 or 8	149902
10	(((prognos* or melanoma) adj5 surviv*) or factor*).ti,ab.	3091009
11	(metas* or advance* or recur* or relaps* or invasive or second* or disseminat*).ti,ab.	3355846
12	(distant metastases or local recurren*).ti,ab.	45561
13	10 or 11 or 12	5901577
14	exp comment/ or exp letter/ or exp editorial/	1731981

#	Searches	Results
15	exp animals/ not exp humans/	4595710
16	(animal or mouse).mp. or mice.ti,ab. [mp=title, abstract, original title, name of substance word, subject heading word, floating sub-heading word, keyword heading word, organism supplementary concept word, protocol supplementary concept word, rare disease supplementary concept word, unique identifier, synonyms]	2072446
17	exp review/	2504343
18	exp case reports/ or case report*.ti,ab.	2103512
19	or/14-18	10922034
20	(3 and 9 and 13) not 19	13880
21	(201808* or 201809* or 20181* or 2019*).ed. or 2019*.dp.	1423830
22	20 and 21	1023

Supplemental Table 2: Updated Medline Search Strategy

Updated search for the period June 2019 to current. Conducted on 30th September 2021

Database(s): Ovid MEDLINE(R) <1946 to September Week 4 2021>

#	Searches	Results
1	Melanoma/	89251
2	melano*.ti,ab,kw.	176833
3	(malignant adj3 melanoma*).ti,ab,kw.	26917
4	skin neoplasms/	127346
5	(tumo* adj5 (mole or melanoma)).ti,ab.	12439
6	1 or 2 or 3 or 4 or 5	271222
7	(ocular or uveal or iris or cornea or eye or choroidal or ciliary or intraocular).ti.	161269
8	6 not 7	262830
9	(metas* or advance* or recur* or relaps* or invasive or second*).ti,ab,kw.	3286319
10	(distant metastases or local recurren*).ti,ab.	45864
11	9 or 10	3286319
12	Cohort Studies/	294363
13	incidence.tw.	705176
14	Mortality/	47593
15	Follow-Up Studies/	671388
16	prognos*.tw.	568943
17	predict*.tw.	1442443
18	course.tw.	523515
19	Survival Analysis/	141766
20	12 or 13 or 14 or 15 or 16 or 17 or 18 or 19	3641777
21	8 and 11 and 20	22371
22	exp case reports/ or case report*.ti,ab,kw.	2112036
23	exp comment/ or exp letter/ or exp editorial/	1835116
24	exp review/	2617551
25	exp animals/ not humans.sh.	4889865
26	22 or 23 or 24 or 25	10795089
27	21 not 26	14321

#	Searches	Results
28	(201906* or 201907* or 201908* or 201909* or 201910* or 201911* or 201912* or "2020" or 2021*).ed. or (2019* or 2020* or 2021*).dp.	2635276
29	27 and 28	2267

Supplemental Table 3: List of databases

- MEDLINE (OVID)
- EMBASE (OVID)
- CENTRAL (Cochrane Library via Wiley)
- Health Technology Assessment database (Cochrane Library via Wiley)
- CINAHL[a] (EBSCO)
- Science Citation Index (Web of Science)
- Conference Proceedings Citation Index – Science (Web of Science)
- Cochrane Database of Systematic Reviews (Cochrane Library via Wiley – to check included studies of relevant reviews).
- Grey literature was sought using similar keywords to search various resources including but not limited to the following:
- OpenGrey (http://www.opengrey.eu/, includes SIGLE, EAGLE, GreyNet)
- Cancer Research UK (http://www.cancerresearchuk.org/about-cancer/find-a-clinical-trial)
- Melanoma UK (http://www.melanomauk.org.uk)
- National Guideline ClearingHouse (https://www.guideline.gov/)
- Ongoing trials identified using the WHO ICTRP platform of trials registries. (www.who.int/trialsearch).

Kunonga TP, *et al. BMJ Open* 2023; 13:e073306. doi: 10.1136/bmjopen-2023-073306

Supplemental Table 4: Definitions for the performance measures

Performance measure	Definition	Statistic/Measure	Presentation	Performance criteria
Discrimination[35,37]	How well a prediction model can discriminate those with the outcome from those without the outcome	C-statistic	ROC curve	Estimates are interpreted as the area under the receiver operating characteristics curve (AUC-ROC), with values between 0.5 (no discriminative ability) to 1 (perfect discriminative ability)
		Discrimination slope	Box plot	
		Harrell C statistic/ concordance statistic	ROC curve	
		Gonen and Heller K-statistic		
Calibration[35,37]	A model's accuracy of predicted risk probabilities and indicates the extent to which expected outcomes (predicted from the model) and observed outcomes agree	Calibration slope	Calibration or validation graph	Calibration can be reported as scores range from 0 (no agreement) to 1 (perfect agreement), or as plots showing the relation between the estimated risk (on the x-axis) and the observed proportion of events (y-axis). A curve close to the diagonal indicates that predicted risks correspond well to observed proportions.
		Calibration-in-the-large	Calibration or validation graph	
		Calibration test	Calibration graph	
		Hosmer-Lemeshow test	Calibration or validation graph	
		Harrell's E Statistic	Calibration graph	
Overall performance[35,37]	The distance between the predicted and observed outcomes	Brier score	Validation graph	Considered as very weak (R2 of 0 to 4%), weak (4 to 16%), moderate (16 to 36%), strong (36 to 64%) or very strong (64% to 100%).
		R-squared statistic	Validation graph	
Clinical utility[44,50]	Defined as the minimum probability of disease at which further intervention would be warranted	Decision curve analysis	Decision curve	Net benefit = sensitivity × prevalence – (1 – specificity) × (1 – prevalence) × w where w is the odds at the threshold probability.
		Net benefit	Cross table	

Kunonga TP, *et al. BMJ Open* 2023; 13:e073306. doi: 10.1136/bmjopen-2023-073306

Supplemental Figure 1: PRISMA Flow Diagram

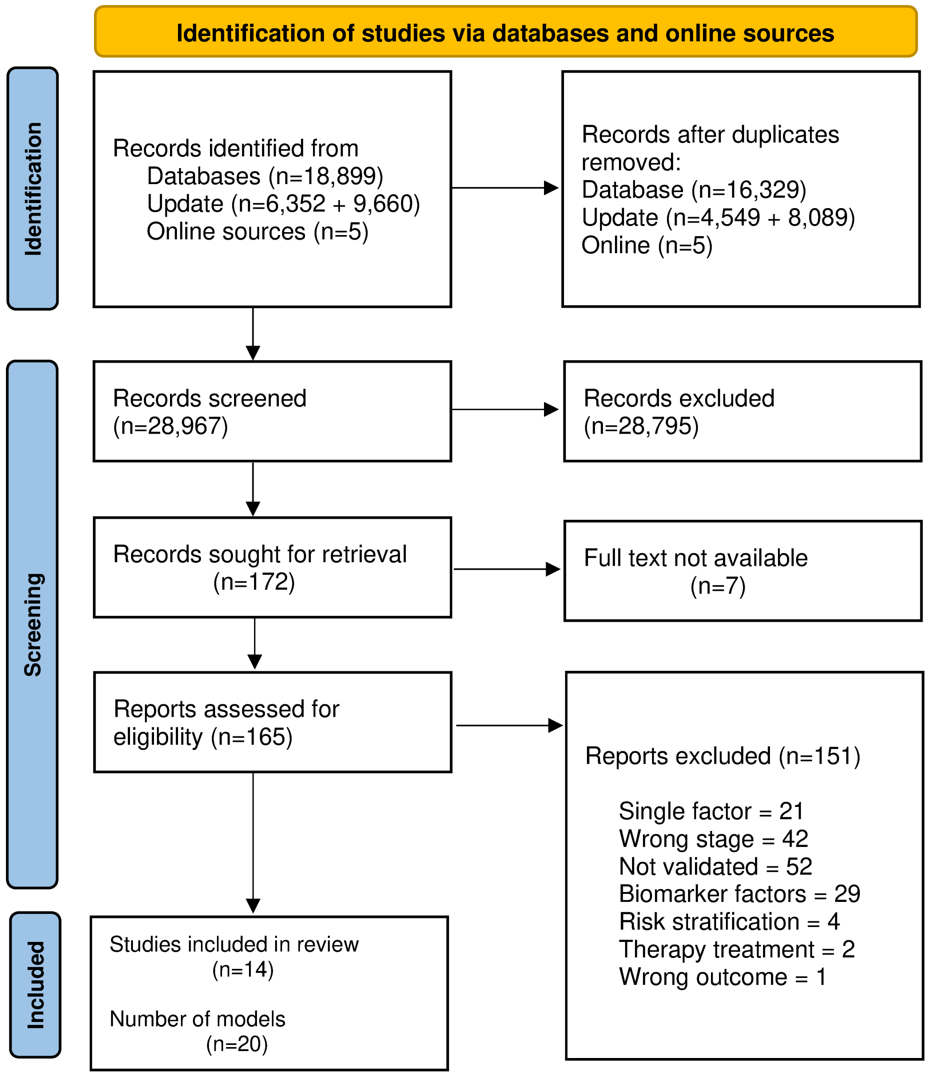

Kunonga TP, *et al. BMJ Open* 2023; 13:e073306. doi: 10.1136/bmjopen-2023-073306

Supplemental Table 5: Model Outcome definitions

Citation	Outcome	Definition provided by authors
Baade 2015[25]	MSS	Melanoma-specific survival was estimated from the mortality of people diagnosed with melanoma between 1995 and 2008 (inclusive), with follow-up for all cases to December 31, 2010.
Balch 2001[26]	MSS	Survival times were calculated from onset of primary melanoma diagnosis and considered censored for patients who were alive at the last follow-up or who died without evidence of melanoma.
Cochran 2000a[27]	OS	Not reported
Cochran 2000b[27]	Recurrence	Not reported
El Sharouni 2021a[28]	LRFS	Recurrence free-survival was calculated from the date of initial melanoma diagnosis to the date of diagnosis of recurrence.
El Sharouni 2021b[28]	RRFS	Recurrence free-survival was calculated from the date of initial melanoma diagnosis to the date of diagnosis of recurrence.
El Sharouni 2021c[28]	DRFS	Recurrence free-survival was calculated from the date of initial melanoma diagnosis to the date of diagnosis of recurrence.
Fonseca 2020a[29]	DFRS	Considered from the date when SNB was performed to the date of the event of interest.
Fonseca 2020b[29]	MSS	Considered from the date when SNB was performed to the date of the event of interest.
Gimotty et al. 2004[31]	Metastasis	Not reported
Gimotty 2007a[30]	OS	Survival time for SEER patients was defined as the time between diagnosis and melanoma-related death
Gimotty 2007b[30]	OS	Survival time for PLG patients was defined as the time between definitive surgical treatment and melanoma-related death.
Maurichi 2014[32]	OS	Overall survival (OS) was calculated from date of surgery for primary melanoma to date of death as a result of all causes or censored at date of last follow-up in living patients.
Rosenbaum 2017[33]	RFS	Recurrence-free survival was defined as the time from the date of pathological diagnosis of stage IB melanoma to the first recorded date of regional or distant metastases
Soong 2010[34]	OS	Survival times were calculated from onset of primary melanoma diagnosis and considered censored for patients who were alive at the last follow-up or who died without evidence of melanoma
Tsai 2007[35]	OS	Not reported

Kunonga TP, *et al. BMJ Open* 2023; 13:e073306. doi: 10.1136/bmjopen-2023-073306

Citation	Outcome	Definition provided by authors
Verver 2019a[36]	Recurrence	Calculated from date of SLNB to date of first recurrence
Verver 2019b[36]	MSM	Calculated from date of SLNB to date of first death
Vollmer 2001[37]	MSS	Not reported
Xiao 2020[38]	OS	Not reported

Abbreviations: ROB = Risk of Bias; MSS = Melanoma Specific Survival; OS = Overall Survival; DRFS = Distant Recurrence-Free survival; RFS = Recurrence Free Survival; RRFS = Regional Recurrence Free Survival; MSM = Melanoma Specific Mortality; SEER = Surveillance, Epidemiology, and End Results Program; PLG = University of Pennsylvania's Pigmented Lesion Group; SLNB = Sentinel Lymph Node Biopsy; SNB = Sentinel Node Biopsy

Supplemental Table 6: Model evaluation

Citation	Predictors in final model	Type of model	Model performance			Validation method
			Discrimination	Calibration	Overall Performance	
Baade 2015[25]	Age, sex, smooth-rank transform thickness, body site, ulceration, positive lymph nodes, metastasis	Melanoma Severity Index	D-statistic = 1.50 (1.44 to 1.56) Harrell-C statistic = 0.88 (0.88 to 0.89)	NA	RD2 statistic = 0.47 (0.45 to 0.49)	Internal-external cross validation
Balch 2001[26]	Age, sex, ulceration, tumour thickness, anatomic level, nodal status, number of metastatic nodes, site	AJCC Validation	NA	NA	NA	External (geographical)
Cochran 2000a[27]	Age, sex, ulceration, site, Breslow thickness	OS Risk Score	NA	NA	NA	Internal (random split)
Cochran 2000b[27]	Ulceration, site, Breslow thickness, melanoma subtype	Recurrence Risk Score	NA	NA	NA	Internal (random split)
El Sharouni 2021a[28]	Age, ulceration, site, Breslow thickness, melanoma subtype, mitosis	LRFS Nomogram	C-statistic DEV: 0.79 (0.76 to 0.82) VALID: 0.80 (0.69 to 0.90)	Calibration plot	NA	Internal (bootstrap) + external (geographical)
El Sharouni 2021b[28]	Sex, ulceration, site, Breslow depth, melanoma subtype, SLN status, mitosis	RRFS Nomogram	C-statistic DEV: 0.77 (0.75 to 0.79) VALID: 0.76 (0.70 to 0.82)	Calibration plot	NA	Internal (bootstrap) + external (geographical)
El Sharouni 2021c[28]	Sex, ulceration, site, Breslow depth, SLN status, mitosis	DRFS Nomogram	C-statistic DEV: 0.80 (0.77 to 0.83) VALID: 0.74 (0.69 to 0.80)	Calibration plot	NA	Internal (bootstrap) + external (geographical)

Kunonga TP, *et al. BMJ Open* 2023; 13:e073306. doi: 10.1136/bmjopen-2023-073306

Citation	Predictors in final model	Type of model	Model performance			Validation method
			Discrimination	Calibration	Overall Performance	
Fonseca 2020a[29]	Age, ulceration, Breslow thickness, mitotic rate, SLN status	DRFS Nomogram	Harrell C-index = 0.734	Calibration plot	NA	Internal (bootstrap)
Fonseca 2020b[29]	Ulceration, Breslow thickness, mitotic rate, SLN status, satellitosis	MSS Nomogram	Harrell C-index = 0.718	Calibration plot	NA	Internal (bootstrap)
Gimotty 2004[31]	Sex, growth phase (vertical or radial), vertical growth phase (VGP), VGP tumour-infiltrating lymphocyte	Metastasis Prognostic classification tree	Risk groups: AUC = 0.85<br>AJCC Stage: AUC = 0.59	NA	NA	External (new cohort)
Gimotty 2007a[30]	Sex, body site, ulceration, tumour thickness, invasion level	OS Prognostic classification tree	SEER: AUC = 0.76	NA	NA	External (geographical)
Gimotty 2007b[30]	Age, sex, clark level, vertical growth mitogenicity	OS Prognostic classification tree	PLG: AUC = 0.83	NA	NA	External (geographical)
Maurichi 2014[32]	Age, ulceration, mitotic rate, lymphovascular invasion, regression, sentinel node status	OS Nomogram	Harrell C statistic = 0.88	Calibration plot	NA	Internal
Rosenbaum 2017[33]	Histopathological width, conformation status (contiguous or noncontiguous), digital or manual area	RFS Nomogram	Youden Index of the AU-ROC curve: AUC = 0.733 (0.647 to 0.818)	NA	NA	Internal (ten-fold cross-validation)
Soong 2010[34]	Age, ulceration, tumour thickness, site, invasion level, lesion site	MSS nomogram<br><br>Web-based tool/ melanomaprognosis.org	NA	Correlation coefficients:<br>5 years = 0.90<br>10 years = 0.93	NA	External (geographical)

Citation	Predictors in final model	Type of model	Model performance			Validation method
			Discrimination	Calibration	Overall Performance	
Tsai 2007[35]	Age, sex, ulceration, tumour thickness, site, invasion level	Prognostic classification tree	NA	NA	Brier score<br>At 1 year: 0.02<br>At 15 years: 0.20	Internal (cross-validation)
Verver 2019a[36]	Ulceration, anatomical location and Breslow thickness	Recurrence Nomogram	C-index = 0.74	Calibration slope 1.10 [0.96 to 1.24]	NA	Internal-external cross validation
Verver 2019b[36]	Ulceration, anatomical location and Breslow thickness	MSM Nomogram	C-index = 0.76		NA	Internal-external cross validation
Vollmer 2001[37]	Age, sex, ulceration, tumour thickness, site	Scoring system	NA	NA	NA	Internal (two-fold cross-validation)
Xiao 2020[38]	Age, sex, race, marital status, anatomic site, stage, depth, ulceration, mitoses, treatment	OS Nomogram	C-index<br>DEV: 0.817 (0.811 to 0.823)<br>VALID: 0.817 (0.809 to 0.825)	Calibration plot	NA	Internal (split sample)

Abbreviations: MSS = Melanoma Specific Survival; OS = Overall Survival; DRFS = Distant Recurrence-Free survival; RFS = Recurrence Free Survival; RRFS = Regional Recurrence Free Survival; AU-ROC = Area under the Receiver Operating Characteristic

Supplemental Table 7: Risk of bias assessment

Study	Endpoint	ROB				Overall R.O.B
		Participants	Predictors	Outcome	Analysis	
Baade 2015[25]	MSS	+	?	+	?	?
Balch 2001[26]	MSS	+	+	+	?	?
Cochran 2000a[27]	OS	-	?	+	-	-
Cochran 2000b[27]	Recurrence	-	?	+	-	-
El Sharouni 2021a[28]	LRFS	+	?	+	+	?
El Sharouni 2021b[28]	RRFS	+	?	+	+	?
El Sharouni 2021c[28]	DRFS	+	?	+	+	?
Fonseca 2020a[29]	DFRS	+	?	+	-	-
Fonseca 2020b[29]	MSS	+	?	+	-	-
Gimotty et al. 2004[31]	Metastasis	+	?	+	+	?
Gimotty 2007a[30]	OS	+	+	+	?	?
Gimotty 2007b[30]	OS	+	+	+	?	?
Maurichi 2014[32]	OS	+	+	+	+	+
Rosenbaum 2017[33]	RFS	+	?	+	-	-
Soong 2010[34]	OS	+	+	+	+	+
Tsai 2007[35]	OS	+	+	+	+	+
Verver 2019a[36]	Recurrence	+	?	+	-	-
Verver 2019b[36]	MSM	+	?	+	-	-
Vollmer 2001[37]	MSM	+	+	+	?	?
Xiao 2020[38]	OS	+	?	+	-	-
+ Indicates low ROB/ low concern regarding applicability						
- Indicates high ROB/ high concern regarding applicability						
? Indicates unclear ROB/ unclear concern regarding applicability						

Abbreviations: ROB = Risk of Bias; MSS = Melanoma Specific Survival; OS = Overall Survival; DRFS = Distant Recurrence-Free survival; RFS = Recurrence Free Survival; RRFS = Regional Recurrence Free Survival; MSM = Melanoma Specific Mortality

Supplemental Table 8: Model Applicability Assessment

Study	Endpoint	Applicability			Overall Applicability
		Participants	Predictors	Outcome	
Baade 2015[25]	MSS	+	+	+	+
Balch 2001[26]	MSS	+	+	+	+
Cochran 2000a[27]	OS	-	+	+	-
Cochran 2000b[27]	Recurrence	-	+	+	-
El Sharouni 2021a[28]	LRFS	+	+	+	+
El Sharouni 2021b[28]	RRFS	+	+	+	+
El Sharouni 2021c[28]	DRFS	+	+	+	+
Fonseca 2020a[29]	DFRS	+	+	+	+
Fonseca 2020b[29]	MSS	+	+	+	+
Gimotty et al. 2004[31]	Metastasis	+	+	+	+
Gimotty 2007a[30]	OS	+	+	+	+
Gimotty 2007b[30]	OS	+	+	+	+
Maurichi 2014[32]	OS	+	+	+	+
Rosenbaum 2017[33]	RFS	+	+	+	+
Soong 2010[34]	OS	+	+	+	+
Tsai 2007[35]	OS	+	+	+	+
Verver 2019a[36]	Recurrence	+	+	+	+
Verver 2019b[36]	MSM	+	+	+	+
Vollmer 2001[37]	MSM	+	+	+	+
Xiao 2020[38]	OS	+	+	+	+
+ Indicates low ROB/ low concern regarding applicability					
- Indicates high ROB/ high concern regarding applicability					
? Indicates unclear ROB/ unclear concern regarding applicability					

Abbreviations: ROB = Risk of Bias; MSS = Melanoma Specific Survival; OS = Overall Survival; DRFS = Distant Recurrence-Free survival; RFS = Recurrence Free Survival; RRFS = Regional Recurrence Free Survival; MSM = Melanoma Specific Mortality

Supplemental File 1: Risk of bias assessment

Assessments were conducted using the PROBAST tool. Overall, eight models were judged to be at high risk of bias, three models were rated as low, and nine models were rated as having an unclear risk. Bias was introduced by various methods.

Selection of participants

Only two models (Cochran OS model,[27] and Cochran recurrence model[27]) were judged to be at high risk of bias. Very little detail was given on the participants in the study and there was no summary of patient characteristics to judge the severity of their melanoma. The rest of the studies were all classified as at low risk of bias.

Risk of bias introduced by predictors

Thirteen models: El Sharouni LRFS model;[28] El Sharouni RRFS model;[28] El Sharouni DRFS model;[28] Cochran OS model;[27] Cochran recurrence model;[27] Fonseca DRFS model;[29] Fonseca MSS model;[29] Verver recurrence model;[36] and Verver MSM model,[36] were rated to have an unclear risk of bias for selection of risk factors included in the final models. The models did not report information on blinding, and the predictors for these models were decided through backward variable selection procedure. With this method, variables are chosen based on significance level, meaning that variables regarded as insignificant, following univariate analysis, are removed from the model. However, the remaining seven models, were rated to have a low risk of bias for this domain. Although the model studies did not report information on blinding of predictor assessment to outcome data, their measurements can be inferred to be blinded to the outcome as predictors appeared to be measured and reported before the outcome occurred.

Risk of bias introduced by outcomes

All models had low concern regarding the timing of assessment. It was felt that the length of follow-up times for the outcomes assessed would not bias the prediction models.

Risk of bias introduced by the analysis

Eight models reported in five studies: Cochran OS model;[27] Cochran recurrence model;[27] Fonseca DRFS model;[29] Fonseca MSS model;[29] Verver recurrence model;[36] Verver MSM model;[36] Rosenbaum RFS model;[33] and Xiao OS model,[38] were judged to be of high risk of bias. This was mainly due to the

Kunonga TP, *et al. BMJ Open* 2023; 13:e073306. doi: 10.1136/bmjopen-2023-073306

inclusion of variables identified as significant following a univariate analysis.[23,29,34] This method can lead to incorrect predictor selection because predictors are chosen on the basis of their statistical significance as a single predictor rather than in context with other predictors.[36,37] Dichotomization of continuous predictors was a concern for some of the models.[25,34] This can potentially reduce a model's predictive ability, by assuming a different risk for different groups.[36,37] Relevant information on whether model overfitting and optimism in model performance was accounted for was not reported in one model.[32]

Concerns regarding applicability

Overall, only the Cochran OS model,[27] and Cochran recurrence model,[27] were rated as having a high-risk regarding applicability, and the remaining nineteen models were rated as having a low risk regarding applicability.

Concern regarding selection of participants

The Cochran OS model,[27] and Cochran recurrence model,[27] were judged to have a high-risk of bias regarding selection of participants. Patients recruited for model development were a subset of the John Wayne Cancer Institute Melanoma database, however not much information was given regarding the characteristics of the patients or the severity of their melanoma. The rest of the models were rated as having a low concern because participant data were taken from routine care or cancer registries, the participants included in the studies matched the participants in the review question. Therefore, the characteristics of patients in the study were likely to match patients with similar characteristics presenting in either a different setting or country.

Concern regarding assessment and timing of predictors

All models had low concern regarding the definition, assessment, and timing of the predictors. All predictors were measured using methods potentially applicable to the daily practice that is addressed by the review.

Concern regarding the applicability of the outcome determined

All models were judged to have a low concern regarding the applicability of the outcomes. Our systematic review explicitly aimed to include all validations of the model regardless of outcome definition and measurement method.
