# Supplementary material for: Predictive accuracy of risk prediction models for recurrence, metastasis and survival for early-stage cutaneous melanoma: a systematic review
Source: BMJ Open. 2023 Sep 28;13(9):e073306. doi: 10.1136/bmjopen-2023-073306 (PMC10546114; doi:10.1136/bmjopen-2023-073306)
Supplement: Author's manuscript [file bmjopen-2023-073306.draft_revisions.pdf]

BMJ Open is committed to open peer review. As part of this commitment we make the peer review history of every article we publish publicly available.

When an article is published we post the peer reviewers' comments and the authors' responses online. We also post the versions of the paper that were used during peer review. These are the versions that the peer review comments apply to.

The versions of the paper that follow are the versions that were submitted during the peer review process. They are not the versions of record or the final published versions. They should not be cited or distributed as the published version of this manuscript.

BMJ Open is an open access journal and the full, final, typeset and author-corrected version of record of the manuscript is available on our site with no access controls, subscription charges or pay-per-view fees (http://bmjopen.bmj.com).

If you have any questions on BMJ Open's open peer review process please email info.bmjopen@bmj.com

BMJ Open

Predictive Accuracy of Risk Prediction Models for Recurrence, Metastasis and Survival for Early-Stage Cutaneous Melanoma: A Systematic Review

Journal:	*BMJ Open*
Manuscript ID	bmjopen-2023-073306
Article Type:	Original research
Date Submitted by the Author:	02-Mar-2023

Complete List of Authors:	Kunonga, Tafadzwa; Newcastle University, Evidence Synthesis Group, Population & Health Sciences Institute; Newcastle University, NIHR Innovation Observatory, Population Health Sciences Institute<br>Kenny, RPW; Newcastle University, Evidence Synthesis Group, Population & Health Sciences Institute; Newcastle University, NIHR Innovation Observatory, Population Health Sciences Institute<br>Astin, Margaret; Newcastle University, Evidence Synthesis Group, Population & Health Sciences Institute<br>Bryant, Andrew; Newcastle University, Biostatistics Research Group, Population Health Sciences Institute<br>Kontogiannis, Vasileios; Newcastle University, Health Economics Group, Population Health Sciences Institute<br>Coughlan, Diarmuid; Newcastle University, Health Economics Group, Population Health Sciences Institute<br>Richmond, Catherine; Newcastle University, Evidence Synthesis Group, Population & Health Sciences Institute; Newcastle University, NIHR Innovation Observatory, Population Health Sciences Institute<br>Eastaugh, Claire H.; Newcastle University, Evidence Synthesis Group, Population & Health Sciences Institute; Newcastle University, NIHR Innovation Observatory, Population Health Sciences Institute<br>Beyer, Fiona ; Newcastle University, Evidence Synthesis Group, Population & Health Sciences Institute; Newcastle University, NIHR Innovation Observatory, Population Health Sciences Institute<br>Pearson, F; Newcastle University, Evidence Synthesis Group, Population & Health Sciences Institute; Newcastle University, NIHR Innovation Observatory, Population Health Sciences Institute<br>Craig, Dawn; Newcastle University, Evidence Synthesis Group, Population & Health Sciences Institute; Newcastle University, NIHR Innovation Observatory, Population Health Sciences Institute<br>Lovat, Penny; Newcastle University, Dermatological Sciences, Translation and Clinical Research Institute; AMLo Biosciences, The Biosphere, Newcastle Helix, Newcastle upon Tyne, NE4 5BX<br>Vale, Luke; Newcastle University, Health Economics Group, Population Health Sciences Institute<br>Ellis, Robert; Newcastle University, Dermatological Sciences, Translational Research Institute; South Tees Hospitals NHS FT, Department of Dermatology

Keywords:	Systematic Review, Adult oncology < ONCOLOGY, Dermatological

	tumours < ONCOLOGY, Epidemiology < ONCOLOGY

SCHOLARONE™
Manuscripts

I, the Submitting Author has the right to grant and does grant on behalf of all authors of the Work (as defined in the below author licence), an exclusive licence and/or a non-exclusive licence for contributions from authors who are: i) UK Crown employees; ii) where BMJ has agreed a CC-BY licence shall apply, and/or iii) in accordance with the terms applicable for US Federal Government officers or employees acting as part of their official duties; on a worldwide, perpetual, irrevocable, royalty-free basis to BMJ Publishing Group Ltd ("BMJ") its licensees and where the relevant Journal is co-owned by BMJ to the co-owners of the Journal, to publish the Work in this journal and any other BMJ products and to exploit all rights, as set out in our licence.

The Submitting Author accepts and understands that any supply made under these terms is made by BMJ to the Submitting Author unless you are acting as an employee on behalf of your employer or a postgraduate student of an affiliated institution which is paying any applicable article publishing charge ("APC") for Open Access articles. Where the Submitting Author wishes to make the Work available on an Open Access basis (and intends to pay the relevant APC), the terms of reuse of such Open Access shall be governed by a Creative Commons licence – details of these licences and which Creative Commons licence will apply to this Work are set out in our licence referred to above.

Other than as permitted in any relevant BMJ Author's Self Archiving Policies, I confirm this Work has not been accepted for publication elsewhere, is not being considered for publication elsewhere and does not duplicate material already published. I confirm all authors consent to publication of this Work and authorise the granting of this licence.

For peer review only

Predictive Accuracy of Risk Prediction Models for Recurrence, Metastasis and Survival for Early-Stage Cutaneous Melanoma: A Systematic Review

Authors

Tafadzwa Patience Kunonga,[1,2] Ryan P.W. Kenny,[1,2] Margaret Astin[1] Andy Bryant,[3] Vasileios Kontogiannis,[4] Diarmuid Coughlan,[4] Catherine Richmond,[1,2] Claire H. Eastaugh,[1,2] Fiona R. Beyer,[1,2] Fiona Pearson,[1,2] Dawn Craig,[1,2,4] Penny Lovat,[5,6] Luke Vale,[4] Robert Ellis,[5,6,7]

1. Evidence Synthesis Group, Population Health Sciences Institute, Newcastle University
2. NIHR Innovation Observatory, Population Health Sciences Institute, Newcastle University
3. Biostatistics Research Group, Population Health Sciences Institute, Newcastle University
4. Health Economics Group, Population Health Sciences Institute, Newcastle University
5. Dermatological Sciences, Translation and Clinical Research Institute, Newcastle University
6. AMLO Biosciences, The Biosphere, Newcastle Helix, Newcastle upon Tyne, NE4 5BX
7. Department of Dermatology, South Tees Hospitals NHS FT

Corresponding author:

Tafadzwa Patience Kunonga, Evidence Synthesis Group, Population Health Sciences Institute, Baddiley Clark Building, Newcastle University, Newcastle upon Tyne NE2 4BN, patience.kunonga@newcastle.ac.uk

Funding/support:

This report was commissioned by the National Institute for Health Research (NIHR) Health Technology Assessment (HTA) Programme (project number 16/166/05) and the NIHR Invention for Innovation (i4i) Innovative Prognostic Test for Early-Stage Cutaneous Melanoma (AMLo) (project reference 20993). The views expressed are those of the author(s) and not necessarily those of the NIHR or the Department of Health and Social Care.

Role of the Funder/Sponsor:

The funding sources had no role in the design and conduct of the study; collection, management, analysis, and interpretation of the data; preparation, review, or approval of the manuscript; and the decision to submit the manuscript for publication.

For peer review only - http://bmjopen.bmj.com/site/about/guidelines.xhtml

Conflict of Interest Disclosures:

Kunonga, Kenny, Astin, Bryant, Kontogiannis, Coughlan, Richmond, Eastaugh, Beyer, Pearson, Nasr have no conflicts of interest to declare. Craig - December 2018 to present, member of the HD&DR Research-led prioritisation committee. Vale was a Member of the NIHR Clinical Evaluation and Trial Panel from 2015-2018. Vale and Craig are both part funded by the NIHR Applied Research Collaboration for the North East and North Cumbria. Lovat is Chief Scientific Officer for AMLo Biosciences Ltd, holds shares in AMLo and is named as an inventor on patents for biomarkers this area. Ellis received personal fees from AMLo Biosciences Ltd, outside the submitted work, he also was previously Chief Medical Officer for AMLo Biosciences Ltd but gave up this position in January 2021. He is named as an inventor on patents owned by AMLo Biosciences Ltd for biomarkers this area.

Data availability statement

All data relevant to the study are included in the article or uploaded as supplemental information.

Word count

Abstract: 246

Manuscript: 3, 367

Tables: 3

Figures: 1

For peer review only - http://bmjopen.bmj.com/site/about/guidelines.xhtml

Abstract

Objectives

To identify studies reporting prognostic models for survival, recurrence and metastasis of melanoma among AJCC stage I and II patients following surgical excision; and determine the performances of these models for predicting relevant clinical outcomes, including overall survival.

Design

A systematic review and narrative synthesis.

Methods

Searches of ten databases were conducted from 2000 to September 2021. Screening, data extraction and risk of bias were undertaken independently by two pairs of reviewers. The diversity of predictors included for model development across the included studies prevented statistical synthesis.

Results

From 28,967 records, fifteen studies reporting twenty models were included; eight (stage I), two (stage II), seven (stages I-II), and three (stages not reported), but were clearly applicable to early stages. Clinicopathologic predictors per model ranged from three to ten. The most common were: ulceration, Breslow thickness/depth, sociodemographic status, and site. Where reported, discriminatory values were ≥ 0.7. Calibration measures showed good matches between predicted and observed rates. None of the studies assessed clinical usefulness of the models. Risk of bias was high in eight models, unclear in nine and low in three. Seven models were internally and externally cross-validated, six models were externally validated, and eight models were internally validated.

Conclusions

All models are effective in their predictive performance, however the low quality of the evidence raises concern as to whether current follow-up recommendations following surgical treatment is adequate. Future models should consider incorporating biomarker factors as they may increase the predictive accuracy of these models.

Key words: early-stage melanoma, prediction model, systematic review, recurrence, survival, metastasis

For peer review only

Article Summary

Strengths and limitations of this study

- Comprehensive and systematic searches captured the core evidence about prediction models in early-stage melanoma.
- The current evidence reviewed across all models is insufficient to make recommendations regarding use in clinical practice.
- Heterogeneity in the data across study populations, predictors, and clinical progression of the disease suggests there is insufficient evidence to make firm conclusions about best clinical practice in the application of predictive models in patients with AJCC stage I or II cutaneous melanoma.

For peer review only - http://bmjopen.bmj.com/site/about/guidelines.xhtml

Introduction

Melanoma is one of the deadliest of all skin cancers.[1] The worldwide incidence of melanoma is estimated to be approximately 2% of cancers per annum.[2] However, in early-stage disease,, once surgically removed from the skin, through wide local excision (WLE), and without adjuvant immunotherapy, chemotherapy or radiotherapy, the majority of early-stage melanomas are cured, with an estimated 5 –year overall survival rate of 95%-100% (stage I), and 65%-92.8% (stage II).[3] However, up to 30% of all primary melanomas progress to metastatic disease with an associated extremely poor survival rate of only 5 – 15%.[4] Although the surgical treatment of primary melanoma is effective and long established, there has been a rapid pace of change recently with the addition of sentinel lymph node biopsy (SLNB).[5] This procedure identifies the sentinel lymph node which is excised for examination to determine whether cancer cells are present.[6]

Currently, a structured, uniformly adopted, evidence-based model of patient follow-up after initial diagnosis and treatment is lacking.[7] Follow-up strategies depend on the stage and progression of the disease; based on the criteria defined within the 8th edition of the American Joint Committee on Cancer (AJCC) staging manual.[8] For this review, however, the definitions of stage and progression used in the 2008, 7th edition[9] and earlier editions remain relevant as the reported data in research studies may be based on definitions from earlier editions. Current guidelines for management of the condition vary across the world, with most developed using anecdotal evidence and expert opinion. A clinical prediction model, a statistical tool that relates multiple factors to the probability of a patient having a future clinical event,[10] could be used to aid early detection of disease progression such as new primary tumours, in-transit metastasis, or locoregional recurrence in the regional lymph nodes. Before introducing a new clinical prediction model in to practice there needs to be evidence of model development, validation and impact. Validation should involve evaluating performance (in terms of discrimination or calibration), and clinical usefulness in practice. Impact should consider whether the model improves decision-making.[10]

Previous systematic reviews have been conducted looking at the value of risk prediction models in primary melanoma.[11-13] However, to our knowledge, no review has critically appraised current clinicopathologic prediction models for primary melanomas (stages I and II), following surgical excision of the tumour. This review aims to identify and assess prognostic/predictive models used to

For peer review only - http://bmjopen.bmj.com/site/about/guidelines.xhtml

predict patient survival, recurrence (any site), and metastasis in AJCC stage (I and II) melanoma following surgical excision, thus potentially allowing further refinement of risk-stratification of patients.

Methods

The study protocol is published on PROSPERO (CRD42018086784). The review population listed in the protocol was later augmented to include stage II melanoma. As with stage I melanoma, tumours at this stage may not have advanced and are treated with WLE.[14] The review followed the Preferred Reporting Items for Systematic Reviews and Meta-Analyses guideline (PRISMA).[15] Searches were originally conducted in July 2019, then updated in September 2021. A date restriction of 2000 was utilised, as this is when SLNB became routine in the USA.[5] The search strategy (Supplemental Table 1 and Supplemental Table 2) was designed in MEDLINE and translated to other databases (Supplemental Table 3). A published and validated prognostic study filter[16] was used including the following concepts: [melanoma] AND [risk models] AND [prognosis]. No language or country of publication restrictions were applied. In the search update (June 2019 to September 2021), a more up to date search filter was used.[17]

Studies were selected using piloted inclusion criteria (Table 1) according to the Patient population, Intervention, Comparator, Outcome, Timing and Setting (PICOTS) formula.[18] The primary outcome is overall survival (OS) defined as patient survival until death from melanoma following primary treatment. Secondary outcomes include: number of detected recurrence and metastasis, and the discrimination, calibration, overall performance, and clinical utility of the models. A table of definitions for the performance measures is presented in Supplemental Table 4. Two reviewers independently screened titles and abstracts for articles relevant to stage I disease. For stage II screening a text mining approach was adopted, followed by duplicate and independent hand screening of study abstracts. Two reviewers independently screened the selected full text articles. For each included study, data were extracted by one of four reviewers using the CHecklist for critical Appraisal and data extraction for systematic Reviews of prediction Modelling Studies (CHARMS) checklist.[19] Data were extracted for characteristics of participants, outcomes, predictors, model development methods, model performance and validation. Extraction forms were checked for accuracy and consistency by another reviewer, and a clinical expert. Each study was assessed for risk of bias by two reviewers independently using the Prediction study Risk of Bias Assessment Tool (PROBAST), addressing four domains that may influence the applicability of the prediction models (participants, predictors,

For peer review only - http://bmjopen.bmj.com/site/about/guidelines.xhtml

outcome and analysis).[20] A narrative synthesis was undertaken, including summarising the characteristics of the included models and the performance of prediction models, focusing on measures of discrimination and calibration, and overall performance; and model validation methods. We were unable to conduct a meta-analysis due to the variance in use of disparate predictors, and modelling methods. Furthermore, it was not possible to perform subgroup analyses as the studies identified did not report outcomes by population subgroups.

Insert Table 1 here

Patient and Public Involvement

This review was requested by our funder within a timescale that did not allow for meaningful public and patient involvement.

Results

Selection of studies

We identified 28,967 records after deduplication, of which 165 were selected for full text screening (Supplemental Figure 1). Fourteen studies reporting twenty unique risk prediction models, met the inclusion criteria.[21-34]

Characteristics of studies

Characteristics of included studies are presented in Table 2. Studies were published between 2000 and 2021; nine from the USA,[22, 23, 26, 27, 29-31, 33, 34] two from Australia,[21, 24] two from Europe,[28, 32] and one from Brazil.[25] Twelve studies were from retrospective cohorts,[21-28, 32-34] and three were from prospective cohorts.[29-31] Patient data were obtained from population-based cancer registries, medical/clinical records or a combination of these sources. Study intervals ranged from 1972 to 2015. Eight models were developed from patients with stage I melanoma only, (three from one study,[24] two from another study,[26] and three from three studies[27-29]), two for stage II only (both reported in the same study[25]), eight for stages I-II (two reported in one study,[32] and five from five studies[21, 22, 30, 31, 34]), and for three models the stages not reported (two from one study,[23] and the other from one study[33]), however they were found to be applicable to stages I-II. Eleven studies used logistic regression

For peer review only - http://bmjopen.bmj.com/site/about/guidelines.xhtml

methods to develop the models,[21-25, 28-30, 32-34] and three used a recursive partitioning method.[26, 27, 31] Duration of follow-up ranged from three[34] to twenty years.[22]

Insert Table 2 here

Characteristics of included models

Characteristics of included models are presented in Table 3. Model outcomes included OS;[21, 23, 26, 28, 30, 31, 34] melanoma specific survival (MSS);[21, 22, 25] melanoma specific mortality (MSM);[32] mortality;[33] recurrence;[23, 32] recurrence free survival (RFS);[29] local RFS (LRFS);[24] regional RFS (RRFS)[24]; distant RFS (DRFS);[24, 25] and metastasis.[27] A table of definitions used in the studies for model outcomes is presented in Supplemental Table 5. Total sample sizes across the models ranged from 259,[25] to 66,192 patients.[34] Five studies selected candidate clinico-pathologic variables based on previous clinical knowledge or literature (pre-specification),[21, 24, 26, 31, 33] three conducted a multivariate analyses,[26, 28, 30] two studies conducted a univariate analysis,[25, 29] while three employed a combination of previous knowledge and univariate analysis.[23, 32, 34] One study did not provide information on variable selection.[22] A backward elimination analysis approach was used in seven studies,[21, 23-25, 27, 29, 32] and a full model approach was used in six studies,[26, 28, 30, 31, 33, 34] to select the final predictors for model development. The number of predictors in the final models ranged from three[29, 32] to ten.[34] The most common predictors were ulceration (17/20), Breslow thickness/depth (16/20), sociodemographic status (15/20), and body/anatomic site (14/20) (See Figure 1). The models were presented as a melanoma severity index,[21] an AJCC validtaion tool,[22] OS risk score,[23] recurrence risk score,[23] LRFS nomogram,[24] RRFS nomogram,[24] two DRFS nomograms,[24, 25] two MSS nomograms,[25, 30] metastasis prognostic classification tree,[27] two OS prognostic classification trees (reported in one study),[26] two OS nomograms,[28, 34] RFS nomogram,[29] prognostic classification tree,[31] recurrence nomogram,[32] MSM nomogram,[32] and a scoring system (mortality rate).[33]

Insert Table 3 here

Insert Figure 1 here

Performance of prediction models

Discrimination

The AUC-ROC was reported in two models (reported in one study) predicting OS,[26] one model predicting RFS,[29] and another predicting metastasis outcomes,[27] all with values above 0.7. The C-index measure was reported in models predicting OS,[21, 28, 34] DRFS,[24, 25] LRFS,[24] RRFS,[24] MSS,[25]

For peer review only - http://bmjopen.bmj.com/site/about/guidelines.xhtml

recurrence,[32] and MSM,[32] all with values above 0.7 for the models. In addition to the C-index, one study also estimated the D-statistic and reported a value of 1.50 (95% CI 1.44 to 1.56) for OS.[21]

Calibration

Calibration scores were reported in one model which reported correlation coefficients of 0.90 and 0.93 for five- and ten-year survival rates.[30] Calibration plots for predicting survival outcomes were presented in ten models predicting OS,[28, 34] DRFS,[24, 25] RRFS,[24] LRFS,[24] MSS,[25] recurrence,[32] and MSM,[32] with results indicating high accuracy and precision of the prediction models, as predicted and actual survival probabilities were similar.[35]

Overall Performance

One model assessed the overall performance of predicting OS by assessing how well the model fit the data using the r-squared (R^2) statistic.[21] The model reported the R^2 as 0.47 (0.45 to 0.49), equating to a strong fit.[21] The other model,[31] measured overall performace by assessing the Brier score, with usual values ranging from 0 (total accuracy) to 1 (wholly innacurate).[36] Results show that the score for the proposed, progonstic classification scheme increased with time from 0.02 at year one to approximately 0.20 at year 15, indicating high accuracy in predicting OS.[31]

Clinical utility

None of the studies conducted either a net benefit or decision curve analysis, therefore we were unable to address the clinical utility of the tools.

Model validation methods

Model validation methods are presented in Supplemental Table 6. Internal validation refers to the performance of a model developed and tested on a sample of the same population.[37] External validation refers to how well a model predicts an outcome in a data set that is different to the development population (new cohort), or a different region or country (geographical).[38] Eight models were internally validated. Two models [29, 33] used the k-fold cross-validation procedure, where an original sample is randomly partitioned into *k* equal-sized subsamples.[39] Three models (two reported in one study,[23] and one in another[34]), randomly selected a proportion of the sample and retained

For peer review only - http://bmjopen.bmj.com/site/about/guidelines.xhtml

these as the validation set, using the rest for training. Maurichi et al.[28] validated their model by assessing the congruence of expected outcomes (predicted from the model) and observed outcomes. Two models reported in one study,[25] were internally validated using bootstrap methods. Bootstrapping is a the process of obtaining average estimates from multiple data sets from an underlying population.[36] Five models validated their models using external techniques, including geographic validation (two reported in one study,[26] and two others[22, 30], and one in new cohorts[27]). Seven models (three reported in one study,[24] two in one study,[32] and two in other studies[21, 31]), assessed model performance using internal-external cross-validation methods (IECV). IECV analyses the performance of models using new patients from different but related practices as compared to the original development sample.[36]

Critical appraisal of included studies

The results of the critical appraisal are presented in Supplemental Table 7. Overall, eight models (two each from three studies,[23, 25, 32] and two from two studies[29, 34]), were judged to be at high risk of bias, three models[28, 30, 31] were rated as low risk, and nine models (three in one study,[24] two in one study,[26] and four in four studies[21, 22, 27, 33]) were rated as having an unclear risk. The most notable cause of high risk was for the analysis domain. This was mainly due to the inclusion of variables in the final model, previously identified as significant following a univariate analysis.[23, 25, 29, 34] This method can lead to incorrect predictor selection because predictors are chosen on the basis of their statistical significance as a single predictor rather than in context with other predictors.[36]

The applicability ratings of the models are presented in Supplemental Table 8. Two models reported in one study,[23] were rated as having a high risk regarding applicability, the rest of the models were rated as low risk of bias regarding applicability. Detailed information on critical appraisal is available in Supplemental File 1. The main concern regarding applicability was for the selection of participants in the study by Cochran et al.,[23] where little information is reported about the characteristics of the patients or the severity of their melanoma.

Discussion

This review identified 14 studies describing 20 different models developed for the prediction of recurrence, new primary tumours or metastasis in patients with AJCC stage I or II cutaneous

For peer review only - http://bmjopen.bmj.com/site/about/guidelines.xhtml

melanoma following excision. The models differed in the predictors used depending on the outcome of interest and statistical measures used to assess model performance. It was therefore inappropriate to statistically synthesise their results. One of the limitations of the studies was the lack of reporting of baseline rates of SLNB, a technique useful for providing disease stage and to guide adjuvant systemic therapy.[40] The absence of these rates makes it difficult to be sure that the patients are correctly classified in the studies. Common risk factors included in the models were consistent with well-established risk factors for melanoma, including ulceration, age, sex, Breslow thickness, and tumour site.[41] Although systematic reviews of prediction models for cutaneous melanoma[12, 13] have previously been conducted, to the best of our knowledge, this review investigates the potential of clinical models to predict recurrence, metastases, and survival of AJCC stage I and II melanoma following surgical excision. Similar to the reviews by Vuong et al.,[13] and Mahar et al.,[11] notable differences in the approaches used to select predictors during model development were observed. Eight models selected predictors based on univariate analysis of the strength of these predictors, of which four were developed based on previous knowledge. When developing models, building on previous literature and expert clinician opinions is recommended. Building models based only on the statistical significance of the association between predictor and outcome in univariate analyses risks missing important predictors.[42]

Model performance measures were available for assessing discrimination in fourteen models, callibration in eleven models, and overall performance in two models. Whilst the type of discrimination statistic varied, the discrimination statistics of the new models ranged from 0.72 to 0.88, a range comparable to that reported in other published melanoma reviews: 0.62 to 0.86,[13] 0.7 to 0.8,[12] and 0.62 to 0.98.[11] The discriminative performance of the models is considered acceptable when the AUC-ROC statistics and their equivalent are ≥ 0.7.[43] Three models that reported callibration scores reported values closer to 1, indicating a perfect agreement, while eight models graphically presented plots showing a good match between predicted and observed outcomes for OS, DRFS, RRFS, LRFS, MSS, and metastasis. This suggests that the models have the ability to accurately generate predictions that are close to the observed outcomes. Currently, there is a lack of evidence for the clinical performance of early stage melanoma prediction models based on clinicopathologic variables. Accuracy measures such as calibration and discrimination, however, do not provide any detail

For peer review only - http://bmjopen.bmj.com/site/about/guidelines.xhtml

regarding a models suitability for clinical practice. Net benefit, obtained via decision curve analysis, aims to assess the clinical utility of such models.[44] Unfortunately, the studies included in this review do not give detail on clinical utility for the models they report upon. In addition, we reviewed follow up schedules as recommended by clinical guidelines and note the limited evidence base on which they are based.[45] This suggests more detailed studies about timing and frequency of relapse are needed. Most of the models were externally validated, either in new cohorts or patients from a different location or through IECV. External validation of models is essential to support the general applicability of any prediction model.[42] However, most of the models were rated as being at high risk of bias implying low confidence in the performance of these models in new datasets.

This review followed procedures documented by the Cochrane Collaboration for conducting systematic reviews, the CHARMS[19] guidelines for extracting data, the PRISMA[15] guidelines for reporting and PROBAST[20] for assessing risk of bias, so was therefore robust. The update search conducted in September 2021 incorporated emerging trial evidence, so we are confident that we would have identified relevant trials/studies coming to light in the next 1-2 years from the search implementation. Having updated this review previously we can also confidently say that the trajectory of research relevant to the review research question is very low. Given this, we are confident that the findings of the review would not change substantively following an update. We had planned to assess the overall quality of evidence using the Grading of Recommendations Assessment, Development and Evaluation (GRADE).[46] However, although the tool has been adapted for assessing overall quality in prognostic factor reviews, studies, it is yet to be adapted for prediction model reviews.[47]

The main limitation was the quality of the published studies. The majority of the studies were published before the reporting gudance for this study design became available, and none of the studies reported having followed the Transparent Reporting of a multivariable prediction model for Individual Prognosis or Diagnosis (TRIPOD)[48] or met the criteria outlined. Secondly, none of the studies reported a decision curve analysis to assess the clinical implications of the models. Thirdly, comparing the included models was problematic and meta-analysis was not feasible due to the variety in the predictors and statistical measures used for model performances. Lastly, we were

For peer review only

unable to access the development and validation studies of some of the publicly available online tools, and these were excluded from our analysis.[49, 50]

The results of this systematic review highlight the relative lack of appropriate evidence underpinning current melanoma prediction tools to support practice in AJCC stage I and II disease. The evidence needed for clinical guideline decision-makers to incorporate a prediction model into routine clinical care needs to be far more convincing than what has been reported thus far. The goal should be clear demonstration of external validity, by evaluating model transportability in other cohorts,[10] and clinical utility (the ability to better select patients from high risk groups for adjuvant therapy in the future) by undertaking decision curve analysis to identify net benefits.[44, 51] We have synthesised evidence on biomarkers, including gene expression profiling (GEP), in additon to clinicopathologic factors to understand if they increase the accuracy of prediction of metastatic risk in SLNB-eligible patients.[52, 53] With a better understanding of melanoma biology, it may be possible to make accurate risk predictions which in turn would result in better response rates for treatments before evidence of recurrence or metastases are detected. However, studies showing the utility of biomarkers predicting high and low risk in early stage melanoma were not included as they were beyond the scope of this review. Additionally, other studies have shown that mutation status is associated with survival outcomes,[54, 55] however we did not evaluate these studies as they were not developed into risk models. Critically, these results clearly outline the need for ongoing prognostication studies and, as such, this review acts as an evidence base to catalyse project development and funding, and future follow-up guidelines and management of patients, given the relative scarcity of evidence-based practice at present.

Conclusion

The data, which are heterogeneous in terms of biology and progression, do not offer a wide enough scope of best practice to allow accurate prognostication of melanoma AJCC stages I or II patients as defined by the criteria within the 3rd, 6th, 7th and 8th editions of AJCC staging, or recommendation for use in clinical practice. This raises concern as to whether current follow-up recommendations post-surgical treatment is adequate as the evidence supporting such recommendation is sparse. Future research should focus on validating existing models utilising TRIPOD guidelines to improve reporting

For peer review only - http://bmjopen.bmj.com/site/about/guidelines.xhtml

quality. Future studies should also look to utilise decision curve analysis to analyse the net benefit of using the predictive model.[51, 56]

Author Contributions:

TPK co-designed the protocol, undertook all stages of the review and co-wrote the paper. RPWK undertook all stages of the review and co-wrote the paper. MA undertook all stages of the review and co-wrote the paper. AB co-designed the protocol undertook all stages of the review and co-wrote the paper, VK undertook all stages of the review and co-wrote the paper. DC undertook all stages of the review and co-wrote the paper. CR undertook the searches for the review and co-wrote the paper. CHE undertook the searches for the review and co-wrote the paper. FRB co-designed the protocol and co-wrote the paper. FP contributed to the analysis of review data and co-wrote the paper. DC co-designed the protocol and co-wrote the paper. PL co-designed the protocol and co-wrote the paper. LV co-designed the protocol and co-wrote the paper. RE co-designed the protocol, undertook all stages of the review and co-wrote the paper.

Acknowledgements:

We would like to thank our colleagues from the Evidence Synthesis Group, and NIHR Innovation Observatory, of Newcastle University and Dr. Batoul Nasr from Dermatological Sciences, Translational Research Institute, Newcastle University for their support in screening.

Data availability statement

All data relevant to the study are included in the article or uploaded as supplemental information.

For peer review only

References

1. Matthews, NH, Li, WQ, Qureshi, AA, et al. Epidemiology of melanoma. In: Ward, WH, Farma, JM, eds. Cutaneous melanoma: etiology and therapy. Brisbane (AU): Codon Publications, 2017.
2. de Vries, E, Sierra, M, Pineros, M, et al. The burden of cutaneous melanoma and status of preventive measures in Central and South America. Cancer Epidemiol 2016;44 Suppl 1:S100-s109.
3. Svedman, FC, Pillas, D, Taylor, A, et al. Stage-specific survival and recurrence in patients with cutaneous malignant melanoma in Europe - a systematic review of the literature. Clin Epidemiol 2016;8:109-122.
4. Balch, CM, Gershenwald, JE, Soong, S-J, et al. Final version of 2009 AJCC melanoma staging and classification. J Clin Oncol 2009;27(36):6199-6206.
5. Thomson, DR, Rughani, MG, Kuo, R, et al. Sentinel node biopsy status is strongly predictive of survival in cutaneous melanoma: Extended follow-up of Oxford patients from 1998 to 2014. J Plast Reconstr Aesthet Surg 2017;70(10):1397-1403.
6. Gershenwald, JE, Ross, MI. Sentinel-lymph-node biopsy for cutaneous melanoma. N Engl J Med 2011;364(18):1738-1745.
7. Barbour, A, Millward, M, Morton, R, et al. Clinical practice guidelines for the diagnosis and management of melanoma 2018. https://wiki.cancer.org.au/australia/Guidelines:Melanoma. Accessed 11 October 2019.
8. American Joint Committee on Cancer. AJCC Cancer Staging Manual. 8th ed. Chicago: American Joint Committee on Cancer/Springer, 2017.
9. American Joint Committee on Cancer. AJCC Cancer Staging Manual. 7th ed. Chicago: American Joint Committee on Cancer, 2010.
10. Moons, KG, Kengne, AP, Grobbee, DE, et al. Risk prediction models: II. External validation, model updating, and impact assessment. Heart 2012;98(9):691-698.
11. Mahar, AL, Compton, C, Halabi, S, et al. Critical assessment of clinical prognostic tools in melanoma. Ann Surg Oncol 2016;23(9):2753-2761.
12. Usher-Smith, JA, Emery, J, Kassianos, AP, et al. Risk prediction models for melanoma: a systematic review. Cancer Epidemiol Biomarkers Prev 2014;23(8):1450-1463.
13. Vuong, K, McGeechan, K, Armstrong, BK, et al. Risk prediction models for incident primary cutaneous melanoma: a systematic review. JAMA Dermatol 2014;150(4):434-444.
14. Cancer Research UK. Treatment for stage 1 and 2 melanoma; 2022. chrome-extension://efaidnbmnnnibpcajpcglclefindmkaj/https://www.nhs.uk/ipgmedia/National/Cancer%20Research%20UK/assets/Treatingearly(Stage1)melanoma(CRUK2pages).pdf. Accessed 21 June 2022.
15. Moher, D, Liberati, A, Tetzlaff, J, et al. Preferred reporting items for systematic reviews and meta-analyses: the PRISMA statement. J Clin Epi 2009;62(10):1006-1012.
16. Geersing, G-J, Bouwmeester, W, Zuithoff, P, et al. Search filters for finding prognostic and diagnostic prediction studies in Medline to enhance systematic reviews. PLoS One 2012;7(2):e32844.
17. Hayden, JA, Wilson, MN, Riley, RD, et al. Individual recovery expectations and prognosis of outcomes in non-specific low back pain: Prognostic factor review. Cochrane Database Syst Rev 2019;(11).
18. Whitlock, EP, Lopez, SA, Chang, S, et al. AHRQ Series Paper 3: Identifying, selecting, and refining topics for comparative effectiveness systematic reviews: AHRQ and the Effective Health-Care program. J Clin Epi 2010;63(5):491-501.
19. Moons, KG, de Groot, JA, Bouwmeester, W, et al. Critical appraisal and data extraction for systematic reviews of prediction modelling studies: the CHARMS checklist. PLoS Med 2014;11(10):e1001744.

For peer review only - http://bmjopen.bmj.com/site/about/guidelines.xhtml

20. Wolff, RF, Moons, KGM, Riley, RD, et al. PROBAST: A tool to assess the risk of bias and applicability of prediction model studies (Prediction model Risk Of Bias ASsessment Tool). Ann Intern Med 2019;170(1):51-58.
21. Baade, PD, Royston, P, Youl, PH, et al. Prognostic survival model for people diagnosed with invasive cutaneous melanoma. BMC Cancer 2015;15:27.
22. Balch, CM, Soong, SJ, Gershenwald, JE, et al. Prognostic factors analysis of 17,600 melanoma patients: Validation of the American Joint Committee on Cancer melanoma staging system. J Clin Oncol 2001;19(16):3622-3634.
23. Cochran, AJ, Elashoff, D, Morton, DL, et al. Individualized prognosis for melanoma patients. Hum Pathol 2000;31(3):327-331.
24. El Sharouni, M-A, Ahmed, T, Varey, AH, et al. Development and validation of nomograms to predict local, regional, and distant recurrence in patients with thin (T1) melanomas. J Clin Oncol 2021;39(11):1243-1252.
25. Fonseca, IB, Lindote, MVN, Monteiro, MR, et al. Sentinel node status is the most important prognostic information for clinical stage IIB and IIC melanoma patients. Ann Surg Oncol 2020;27(11):4133-4140.
26. Gimotty, PA, Elder, DE, Fraker, DL, et al. Identification of high-risk patients among those diagnosed with thin cutaneous melanomas. J Clin Oncol 2007;25(9):1129-1134.
27. Gimotty, PA, Guerry, D, Ming, ME, et al. Thin primary cutaneous malignant melanoma: a prognostic tree for 10-year metastasis is more accurate than American Joint Committee on Cancer staging. J Clin Oncol 2004;22(18):3668-3676.
28. Maurichi, A, Miceli, R, Camerini, T, et al. Prediction of survival in patients with thin melanoma: results from a multi-institution study. J Clin Oncol 2014;32(23):2479-2485.
29. Rosenbaum, BE, Schafer, C, Han, SW, et al. Computer-assisted image analysis demonstrates tumor area and width as prognostic factors in stage IB melanoma. Pigment Cell Melanoma Res 2017;30(1):138.
30. Soong, SJ, Ding, S, Coit, D, et al. Predicting survival outcome of localized melanoma: An electronic prediction tool based on the AJCC melanoma database. Ann Surg Oncol 2010;17(8):2006-2014.
31. Tsai, CA, Chen, DT, Chen, JJ, et al. An integrated tree-based classification approach to prognostic grouping with application to localized melanoma patients. J Biopharm Stat 2007;17(3):445-460.
32. Verver, D, van Klaveren, D, Franke, V, et al. Development and validation of a nomogram to predict recurrence and melanoma-specific mortality in patients with negative sentinel lymph nodes. Journal of British Surgery 2019;106(3):217-225.
33. Vollmer, RT, Seigler, HF. Using a continuous transformation of the breslow thickness for prognosis in cutaneous melanoma. Am J Clin Pathol 2001;115(2):205-212.
34. Xiao, Y, Peng, S, Hu, Y, et al. Development and validation of prognostic nomogram in patients with nonmetastatic malignant melanoma: a SEER population-based study. Cancer Medicine 2020;9(22):8562-8570.
35. Austin, PC, Steyerberg, EW. Graphical assessment of internal and external calibration of logistic regression models by using loess smoothers. Stat Med 2014;33(3):517-535.
36. Steyerberg, EW. Clinical prediction models. New York: Springer, 2009.
37. Steyerberg, EW, Harrell Jr, FE, Borsboom, GJ, et al. Internal validation of predictive models: efficiency of some procedures for logistic regression analysis. J Clin Epi 2001;54(8):774-781.
38. Debray, TP, Vergouwe, Y, Koffijberg, H, et al. A new framework to enhance the interpretation of external validation studies of clinical prediction models. J Clin Epi 2015;68(3):279-289.
39. Wong, T-T. Performance evaluation of classification algorithms by k-fold and leave-one-out cross validation. Pattern Recognition 2015;48(9):2839-2846.

For peer review only - http://bmjopen.bmj.com/site/about/guidelines.xhtml

40. Johansson, I, Tempel, D, Dwarkasing, J, et al. Validation of a clinicopathological and gene expression profile model to identify patients with cutaneous melanoma where sentinel lymph node biopsy is unnecessary. Eur J Surg Oncol 2022;48(2):320-325.
41. Buja, A, Bardin, A, Damiani, G, et al. Prognosis for Cutaneous Melanoma by Clinical and Pathological Profile: A Population-Based Study. Frontiers in Oncology 2021;11:737399-737399.
42. Steyerberg, EW, Vickers, AJ, Cook, NR, et al. Assessing the performance of prediction models: a framework for some traditional and novel measures. Epidemiology 2010;21(1):128.
43. Debray, TP, Koffijberg, H, Nieboer, D, et al. Meta-analysis and aggregation of multiple published prediction models. Stat Med 2014;33(14):2341-2362.
44. Vickers, AJ, Van Calster, B, Steyerberg, EW. Net benefit approaches to the evaluation of prediction models, molecular markers, and diagnostic tests. BMJ 2016;352.
45. Vale, L, Kunonga, P, Coughlan, D, et al. Optimal surveillance strategies for patients with stage 1 cutaneous melanoma post primary tumour excision: three systematic reviews and an economic model. Health Technology Assessment 2021.
46. Schünemann, H, Brożek, J, Guyatt, G, et al. GRADE handbook for grading quality of evidence and strength of recommendations; 2013. https://gdt.gradepro.org/app/handbook/handbook.html. Accessed 18 December 2020.
47. Iorio, A, Spencer, FA, Falavigna, M, et al. Use of GRADE for assessment of evidence about prognosis: rating confidence in estimates of event rates in broad categories of patients. BMJ 2015;350:h870.
48. Collins, GS, Reitsma, JB, Altman, DG, et al. Transparent reporting of a multivariable prediction model for individual prognosis or diagnosis (TRIPOD): the TRIPOD statement. BMC Med 2015;13(1):1.
49. Michaelson, J. Melanoma Conditional Outcome Calculator; 2011. http://lifemath.net/cancer/melanoma/condsurv/index.php. Accessed 13 June 2022.
50. Michaelson, J. Melanoma Outcome Calculator; 2011. http://lifemath.net/cancer/melanoma/outcome/index.php. Accessed 13 June 2022.
51. Vickers, AJ, van Calster, B, Steyerberg, EW. A simple, step-by-step guide to interpreting decision curve analysis. Diagn Progn Res 2019;3(1):1-8.
52. Gerami, P, Cook, RW, Russell, MC, et al. Gene expression profiling for molecular staging of cutaneous melanoma in patients undergoing sentinel lymph node biopsy. J Am Acad Dermatol 2015;72(5):780-785. e783.
53. Sominidi-Damodaran, S, Pittelkow, MR, Meves, A. Gene expression profiling in cutaneous melanoma: caveats for clinicians. *Mayo Clinic Proceedings.* 91. Elsevier; 2016:1147-1148.
54. Adler, NR, Wolfe, R, Kelly, JW, et al. Tumour mutation status and sites of metastasis in patients with cutaneous melanoma. Br J Cancer 2017;117(7):1026-1035.
55. Calomarde-Rees, L, García-Calatayud, R, Caballero, CR, et al. Risk factors for lymphatic and hematogenous dissemination in patients with stages I to II cutaneous melanoma. JAMA Dermatol 2019;155(6):679-687.
56. Sadatsafavi, M, Adibi, A, Puhan, M, et al. Moving beyond AUC: decision curve analysis for quantifying net benefit of risk prediction models. European Respiratory Journal 2021;58(5).

For peer review only - http://bmjopen.bmj.com/site/about/guidelines.xhtml

Table 1: Inclusion criteria

Criteria	Include	Exclude
Population	Adults aged ≥ 18 years treated for American Joint Committee on Cancer (7th Edition) stage I and II cutaneous melanoma<br>➢ stage IA (T1a ≤ 1mm thick); or<br>➢ stage IB (T1b with ulceration or mitoses ≤ 1 mm thick, or T2a 1.01 to 2.00mm thick and no ulceration); or<br>➢ stage IIA (1.01-2.00mm thick with ulceration, or 2.01-4.00mm thick without ulceration); or<br>➢ stage IIB (2.01-4.00mm thick with ulceration, or > 4.0mm without ulceration)<br>➢ stage IIC (>4.0mm with ulceration)	Advanced melanoma<br>Metastatic melanoma<br>Stage III<br>Stage IV
Types of prognostic models	➢ Nomogram<br>➢ Scoring system<br>➢ Equation<br>➢ Classification or decision trees<br>*Minimum of 2 clinicopathologic factors	Models built with predictive biomarker factors included<br>Models built with gene expression profiling<br>Diagnostic studies<br>Studies assessing only one predictor
Outcome measures	Primary: Overall survival<br>Secondary: recurrence (any site), metastases, prognostic performance of risk models	
Timing	Post-resection of the primary cutaneous tumour<br>➢ Diagnostic excision<br>➢ Re-excision, also called wide local excision (WLE)	Studies that are looking at treatment (e.g. therapy) of melanoma
Setting	Any setting (primary, secondary, or tertiary care)	
Study design	Retrospective cohort studies<br>Prospective cohort studies<br>Randomised controlled trials	Non-empirical studies<br>Non-English papers that we cannot translate

For peer review only

Table 2: Characteristics of included studies

Citation	Study location	Study design	Data source (no. of centres)	AJCC Disease stage/Clinical staging	Study interval	Development method
Baade 2015[21]	Australia	Retrospective	Population based Queensland cancer registry	2002 AJCC<br>94% Stages I-II	1995 - 2008	Multivariate analysis
Balch 2001[22]	USA	Retrospective	Population based AJCC database (13)	Stages I-II	Not reported	Multivariate analysis
Cochran 2000[23]	USA	Retrospective	John Wayne Cancer Institute Melanoma clinical database, Division of Surgical Oncology, UCLA	1988 AJCC<br>Not reported	1980 - 1990	Multivariate analysis
El Sharouni 2021[24]	Netherlands<br>Australia	Retrospective	DEV: PALGA, the Dutch Pathology Registry<br>VALID: Melanoma institute Australia	2009 AJCC<br>Stages IA-IB<br>IA (T1a) = 58.6%<br>IB (T1b) = 29%<br>T1nos = 12.4%	2000 - 2014	Multivariate analysis
Fonseca 2020[25]	Brazil	Retrospective	A. C. Camargo Cancer Center database	2009 AJCC<br>Stages IIB-IIC	2000 - 2014	Multivariate analysis
Gimotty 2004[27]	USA	Retrospective	DEV: SEER population based registry<br>VALID: New SEER patients	2002 AJCC<br>Stages IA-IB<br>IA (T1a) = 86%<br>IB (T1b) = 14%	DEV: 1972 - 1991<br>VALID: 1991 - 1995	Recursive partitioning
Gimotty 2007[26]	USA	Retrospective	DEV: SEER Registry<br>VALID: Clinical based PLC registry	2002 AJCC<br>Stages IA (T1a) –IB (T1b)	DEV: 1998 – 2001<br>VALID: 1972 - 2001	Recursive partitioning
Maurichi 2014[28]	Europe	Retrospective	European clinical based centres (6)	2009 AJCC<br>Stages IA-IB<br>T1a = 50.3%<br>T1b = 49.7%	1996 - 2004	Multivariate analysis
Rosenbaum 2017[29]	USA	Prospective	NYU clinico-pathological biospecimen database	2009 AJCC<br>Stage IB	2002 - 2014	Multivariate analysis
Soong 2010[30]	USA	Prospective	DEV: 2008 AJCC population based Melanoma Database (9)<br>VALID: Sydney Melanoma Unit, Australia	2008 AJCC<br>Stages I-II	DEV: 26% diagnosed after 2002<br>VALID: Not reported	Multivariate analysis
Tsai 2007[31]	USA	Prospective	AJCC Melanoma population-based database (13)	2002 AJCC<br>Stages IA-IIC	Not reported	Recursive partitioning

For peer review only

Citation	Study location	Study design	Data source (no. of centres)	AJCC Disease stage/Clinical staging	Study interval	Development method
Verver 2019[32]	Europe	Retrospective	EORTC	2009 AJCC Stages IA-IIC	1997 - 2013	Multivariate analysis
Vollmer 2001[33]	USA	Retrospective	University Melanoma Clinic database	1988 AJCC Not reported	1980 - 1990	Multivariate analysis
Xiao 2020[34]	USA	Retrospective	SEER Registry	2009 AJCC Stages IA-IIC	2010 - 2015	Multivariate analysis

Abbreviations: AJCC = American Joint Committee on Cancer; DEV = Development set; NYU = New York University; EORTC = European Organization for Research and Treatment of Cancer Centres SEER = Surveillance, Epidemiology, and End Results Program; UCLA = University of California, Los Angeles; VALID = Validation Set; RND = Regional Node Dissection

For peer review only

Table 3: Model development

Citation	Sample size	Follow up period	End point	Number of events	Candidate selection method	Final predictor selection	Number of final predictors
Baade 2015[21]	28,654	Median = 7.2 years (86.4 months)	MSS	1,700	Pre-specification from existing data	Backward elimination	7
Balch 2001[22]	13,581	5 - 20 years	MSS	NR	Unclear	Unclear	8
Cochran 2000a[23]	1,042	Median = 42.5 months	OS	NR	Pre-specification + univariate analysis	Backward elimination	5
Cochran 2000b[23]	1042	Median = 42.5 months	Recurrence	NR	Pre-specification + univariate analysis	Backward elimination	4
El Sharouni 2021a[24]	DEV: 25,930 VALID: 2,968	DEV: Median = 6.7yrs VALID: Median = 12yrs	LRFS	232	Pre-specification from existing data	Backward elimination	6
El Sharouni 2021b[24]	DEV: 25,930 VALID: 2,968	DEV: Median = 6.7yrs VALID: Median = 12yrs	RRFS	564	Pre-specification from existing data	Backward elimination	7
El Sharouni 2021c[24]	DEV: 25,930 VALID: 2,968	DEV: Median = 6.7yrs VALID: Median = 12yrs	DRFS	278	Pre-specification from existing data	Backward elimination	6
Fonseca 2020a[25]	259	Median = 80.13 months	DRFS	117	Univariate analysis	Backward elimination	5
Fonseca 2020b[25]	259	Median = 80.13 months	MSS	117	Univariate analysis	Backward elimination	5
Gimott y 2004[27]	DEV: 884 VALID: 114	At least 10 years	Metastasis	127	Multivariate analysis	Backward elimination	4
Gimotty 2007a[26]	DEV: 26,114	Median = 4.6 years	OS	3,593	Pre-specification from existing data	Full model approach	4

For peer review only - http://bmjopen.bmj.com/site/about/guidelines.xhtml

Citation	Sample size	Follow up period	End point	Number of events	Candidate selection method	Final predictor selection	Number of final predictors
Gimotty 2007b[26]	VALID: 2,389	Median = 8.1 years	OS	1076	Pre-specification from existing data	Full model approach	5
Maurichi 2014[28]	2,243	124 months	12-year OS	240	Multivariate analysis	Full model approach	6
Rosenbaum 2017[29]	DEV: 506 VALID: 149	Median = 4.4 years	RFS	NR	Univariate analysis	Backward elimination	3
Soong 2010[30]	DEV: 14,760 VALID: 10,974	5 and 10 years	OS	NR	Multivariate analysis	Full model approach	6
Tsai 2007[31]	13,268	Not reported	OS	NR	Pre-specification from existing data	Full model approach	6
Verver 2019[32]	3180	Median = 61 months	Composite Recurrence and/or MSM	Recurrence = 496 Dead = 277	Pre-specification + univariate analysis	Backward elimination	3
Vollmer 2001[33]	1,910	Median = 7.6 years	Mortality	NR	Pre-specification	Full model approach	5
Xiao 2020[34]	DEV: 46,336 VALID: 19,856	3 and 5 years	OS	NR	Pre-specification + univariate analysis	Full model approach	10

Abbreviations: DEV = Development set; VALID = Validation set; MSS = Melanoma Specific Survival; OS = Overall Survival; DRFS = Distant Recurrence-Free survival; RFS = Recurrence Free Survival; RRFS = Regional Recurrence Free Survival; NR = Not Reported; EPV = Events Per Variable

For peer review only - http://bmjopen.bmj.com/site/about/guidelines.xhtml

Supplementary Materials

Supplemental Table 1: Medline Search Strategy

Supplemental Table 2: Updated Medline Search Strategy

Supplemental Table 3: List of databases

Supplemental Table 4: Definitions for the performance measures

Supplemental Figure 1: PRISMA Flow Diagram

Supplemental Table 5: Model outcome definitions

Supplemental Table 6: Model evaluation

Supplemental Table 7: Risk of bias assessment

Supplemental Table 8: Model Applicability Assessment

Supplemental File 1: Risk of bias assessment

PRISMA Checklist

For peer review only

For peer review only - http://bmjopen.bmj.com/site/about/guidelines.xhtml

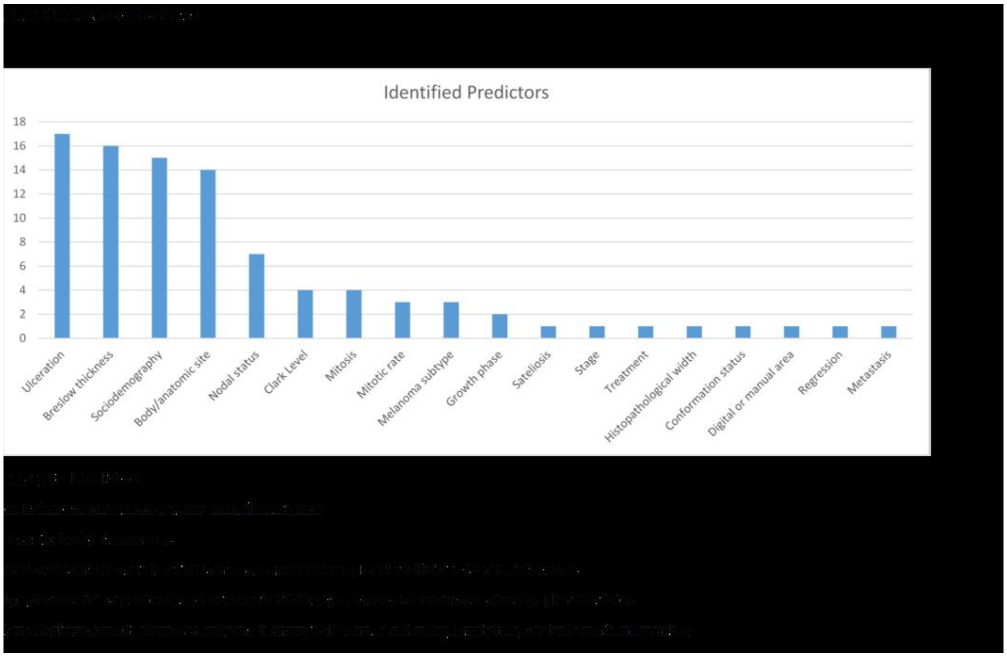

Identified Predictors

89x58mm (300 x 300 DPI)

Supplementary Materials

Supplemental Table 1: Medline Search Strategy

Originally searched September 2018, update searches in 16th July 2019, and 30th September 2021

Database(s): Ovid MEDLINE(R) and In-Process & Other Non-Indexed Citations 1946 to July 15, 2019

#	Searches	Results
1	(Validat* or Predict* or Rule* or (Predict* and (Outcome* or Risk* or Model*)) or ((History or Variable* or Criteria or Scor* or Characteristic* or Finding* or Factor*) and (Predict* or Model* or Decision* or Identif* or Prognos*))).mp. or (Decision*.mp. and ((Model* or Clinical*).mp. or Logistic Models/)) or (Prognostic and (History or Variable* or Criteria or Scor* or Characteristic* or Finding* or Factor* or Model*)).ti,ab. [mp=title, abstract, original title, name of substance word, subject heading word, floating sub-heading word, keyword heading word, organism supplementary concept word, protocol supplementary concept word, rare disease supplementary concept word, unique identifier, synonyms]	4600871
2	Stratification.mp. or ROC Curve/ or Discrimination.mp. or Discriminate.mp. or c-statistic.mp. or c statistic.mp. or Area under the curve.mp. or AUC.mp. or Calibration.mp. or Indices.mp. or Algorithm.mp. or Multivariable.mp. or Prognosis.mp. [mp=title, abstract, original title, name of substance word, subject heading word, floating sub-heading word, keyword heading word, organism supplementary concept word, protocol supplementary concept word, rare disease supplementary concept word, unique identifier, synonyms]	1450061
3	1 or 2	5285509
4	*melanoma/	65466
5	*skin neoplasms/	99710
6	melanoma.kw.	7937
7	(malignant adj3 melanoma*).ti,ab.	27244
8	(tumo* adj5 (mole or melanoma)).ti,ab.	12160
9	4 or 5 or 6 or 7 or 8	149902
10	(((prognos* or melanoma) adj5 surviv*) or factor*).ti,ab.	3091009
11	(metas* or advance* or recur* or relaps* or invasive or second* or disseminat*).ti,ab.	3355846
12	(distant metastases or local recurren*).ti,ab.	45561
13	10 or 11 or 12	5901577
14	exp comment/ or exp letter/ or exp editorial/	1731981

For peer review only - http://bmjopen.bmj.com/site/about/guidelines.xhtml

#	Searches	Results
15	exp animals/ not exp humans/	4595710
16	(animal or mouse).mp. or mice.ti,ab. [mp=title, abstract, original title, name of substance word, subject heading word, floating sub-heading word, keyword heading word, organism supplementary concept word, protocol supplementary concept word, rare disease supplementary concept word, unique identifier, synonyms]	2072446
17	exp review/	2504343
18	exp case reports/ or case report*.ti,ab.	2103512
19	or/14-18	10922034
20	(3 and 9 and 13) not 19	13880
21	(201808* or 201809* or 20181* or 2019*).ed. or 2019*.dp.	1423830
22	20 and 21	1023

For peer review only

Supplemental Table 2: Updated Medline Search Strategy

Updated search for the period June 2019 to current. Conducted on 30th September 2021

Database(s): Ovid MEDLINE(R) <1946 to September Week 4 2021>

#	Searches	Results
1	Melanoma/	89251
2	melano*.ti,ab,kw.	176833
3	(malignant adj3 melanoma*).ti,ab,kw.	26917
4	skin neoplasms/	127346
5	(tumo* adj5 (mole or melanoma)).ti,ab.	12439
6	1 or 2 or 3 or 4 or 5	271222
7	(ocular or uveal or iris or cornea or eye or choroidal or ciliary or intraocular).ti.	161269
8	6 not 7	262830
9	(metas* or advance* or recur* or relaps* or invasive or second*).ti,ab,kw.	3286319
10	(distant metastases or local recurren*).ti,ab.	45864
11	9 or 10	3286319
12	Cohort Studies/	294363
13	incidence.tw.	705176
14	Mortality/	47593
15	Follow-Up Studies/	671388
16	prognos*.tw.	568943
17	predict*.tw.	1442443
18	course.tw.	523515
19	Survival Analysis/	141766
20	12 or 13 or 14 or 15 or 16 or 17 or 18 or 19	3641777
21	8 and 11 and 20	22371
22	exp case reports/ or case report*.ti,ab,kw.	2112036
23	exp comment/ or exp letter/ or exp editorial/	1835116
24	exp review/	2617551
25	exp animals/ not humans.sh.	4889865
26	22 or 23 or 24 or 25	10795089
27	21 not 26	14321

For peer review only

#	Searches	Results
28	(201906* or 201907* or 201908* or 201909* or 201910* or 201911* or 201912* or "2020" or 2021*).ed. or (2019* or 2020* or 2021*).dp.	2635276
29	27 and 28	2267

For peer review only

Supplemental Table 3: List of databases

- MEDLINE (OVID)
- EMBASE (OVID)
- CENTRAL (Cochrane Library via Wiley)
- Health Technology Assessment database (Cochrane Library via Wiley)
- CINAHL[a] (EBSCO)
- Science Citation Index (Web of Science)
- Conference Proceedings Citation Index – Science (Web of Science)
- Cochrane Database of Systematic Reviews (Cochrane Library via Wiley – to check included studies of relevant reviews).
- Grey literature was sought using similar keywords to search various resources including but not limited to the following:
- OpenGrey (http://www.opengrey.eu/, includes SIGLE, EAGLE, GreyNet)
- Cancer Research UK (http://www.cancerresearchuk.org/about-cancer/find-a-clinical-trial)
- Melanoma UK (http://www.melanomauk.org.uk)
- National Guideline ClearingHouse (https://www.guideline.gov/)
- Ongoing trials identified using the WHO ICTRP platform of trials registries. (www.who.int/trialsearch).

For peer review only - http://bmjopen.bmj.com/site/about/guidelines.xhtml

Supplemental Table 4: Definitions for the performance measures

Performance measure	Definition	Statistic/Measure	Presentation	Performance criteria
Discrimination[35,37]	How well a prediction model can discriminate those with the outcome from those without the outcome	C-statistic	ROC curve	Estimates are interpreted as the area under the receiver operating characteristics curve (AUC-ROC), with values between 0.5 (no discriminative ability) to 1 (perfect discriminative ability)
		Discrimination slope	Box plot	
		Harrell C statistic/ concordance statistic	ROC curve	
		Gonen and Heller K-statistic		
Calibration[35,37]	A model's accuracy of predicted risk probabilities and indicates the extent to which expected outcomes (predicted from the model) and observed outcomes agree	Calibration slope	Calibration or validation graph	Calibration can be reported as scores range from 0 (no agreement) to 1 (perfect agreement), or as plots showing the relation between the estimated risk (on the x-axis) and the observed proportion of events (y-axis). A curve close to the diagonal indicates that predicted risks correspond well to observed proportions.
		Calibration-in-the-large	Calibration or validation graph	
		Calibration test	Calibration graph	
		Hosmer-Lemeshow test	Calibration or validation graph	
		Harrell's E Statistic	Calibration graph	
Overall performance[35,37]	The distance between the predicted and observed outcomes	Brier score	Validation graph	Considered as very weak (R2 of 0 to 4%), weak (4 to 16%), moderate (16 to 36%), strong (36 to 64%) or very strong (64% to 100%).
		R-squared statistic	Validation graph	
Clinical utility[44,50]	Defined as the minimum probability of disease at which further intervention would be warranted	Decision curve analysis	Decision curve	Net benefit = sensitivity × prevalence – (1 – specificity) × (1 – prevalence) × w where w is the odds at the threshold probability.
		Net benefit	Cross table	

For peer review only

For peer review only - http://bmjopen.bmj.com/site/about/guidelines.xhtml

Supplemental Figure 1: PRISMA Flow Diagram

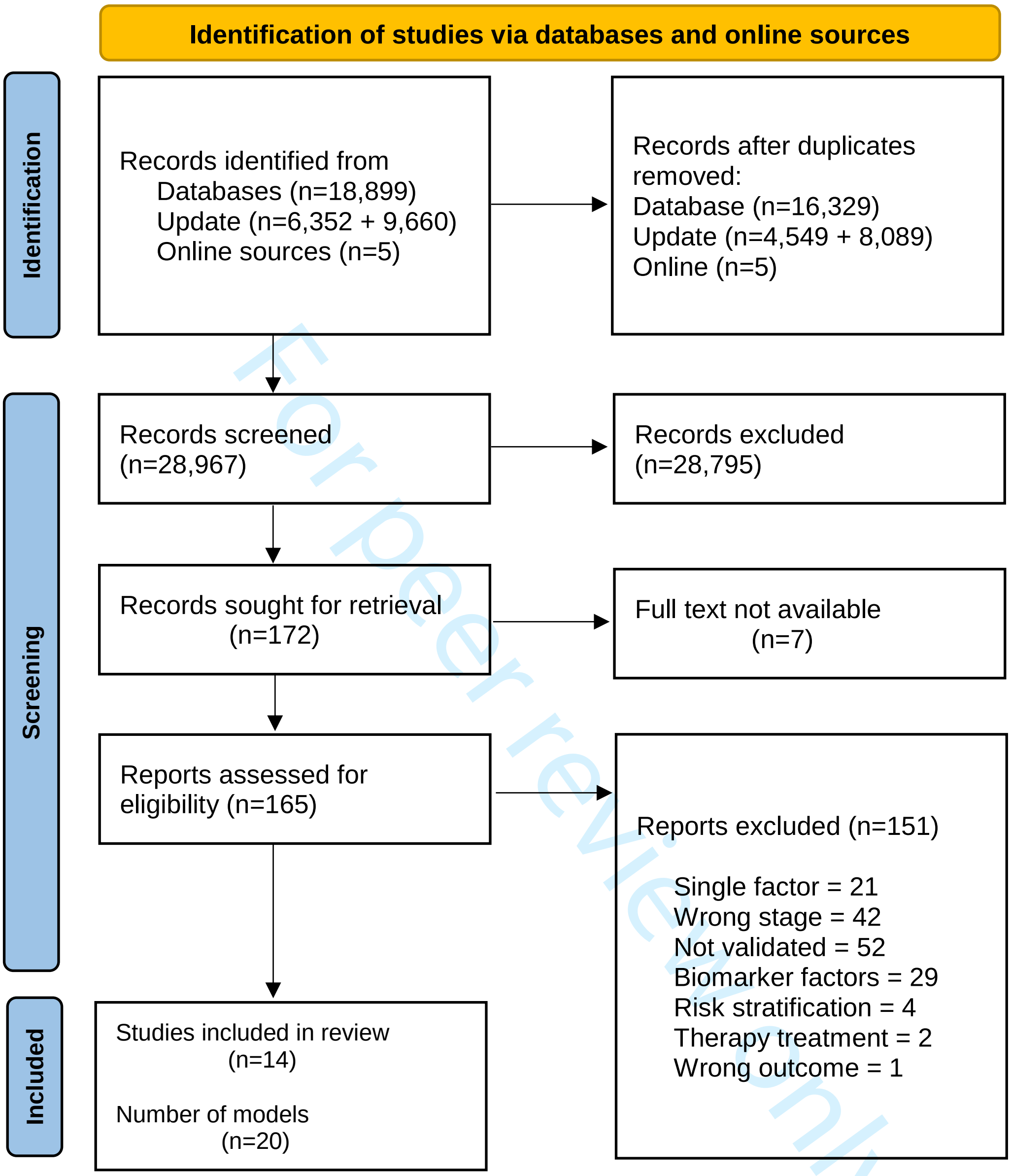

For peer review only - http://bmjopen.bmj.com/site/about/guidelines.xhtml

Supplemental Table 5: Model Outcome definitions

Citation	Outcome	Definition provided by authors
Baade 2015[21]	MSS	Melanoma-specific survival was estimated from the mortality of people diagnosed with melanoma between 1995 and 2008 (inclusive), with follow-up for all cases to December 31, 2010.
Balch 2001[22]	MSS	Survival times were calculated from onset of primary melanoma diagnosis and considered censored for patients who were alive at the last follow-up or who died without evidence of melanoma.
Cochran 2000a[23]	OS	Not reported
Cochran 2000b[23]	Recurrence	Not reported
El Sharouni 2021a[24]	LRFS	Recurrence free-survival was calculated from the date of initial melanoma diagnosis to the date of diagnosis of recurrence.
El Sharouni 2021b[24]	RRFS	Recurrence free-survival was calculated from the date of initial melanoma diagnosis to the date of diagnosis of recurrence.
El Sharouni 2021c[24]	DRFS	Recurrence free-survival was calculated from the date of initial melanoma diagnosis to the date of diagnosis of recurrence.
Fonseca 2020a[25]	DFRS	Considered from the date when SNB was performed to the date of the event of interest.
Fonseca 2020b[25]	MSS	Considered from the date when SNB was performed to the date of the event of interest.
Gimotty et al. 2004[27]	Metastasis	Not reported
Gimotty 2007a[26]	OS	Survival time for SEER patients was defined as the time between diagnosis and melanoma-related death
Gimotty 2007b[26]	OS	Survival time for PLG patients was defined as the time between definitive surgical treatment and melanoma-related death.
Maurichi 2014[28]	OS	Overall survival (OS) was calculated from date of surgery for primary melanoma to date of death as a result of all causes or censored at date of last follow-up in living patients.
Rosenbaum 2017[29]	RFS	Recurrence-free survival was defined as the time from the date of pathological diagnosis of stage IB melanoma to the first recorded date of regional or distant metastases
Soong 2010[30]	OS	Survival times were calculated from onset of primary melanoma diagnosis and considered censored for patients who were alive at the last follow-up or who died without evidence of melanoma
Tsai 2007[31]	OS	Not reported

For peer review only - http://bmjopen.bmj.com/site/about/guidelines.xhtml

Citation	Outcome	Definition provided by authors
Verver 2019a[32]	Recurrence	Calculated from date of SLNB to date of first recurrence
Verver 2019b[32]	MSM	Calculated from date of SLNB to date of first death
Vollmer 2001[33]	MSS	Not reported
Xiao 2020[34]	OS	Not reported

Abbreviations: ROB = Risk of Bias; MSS = Melanoma Specific Survival; OS = Overall Survival; DRFS = Distant Recurrence-Free survival; RFS = Recurrence Free Survival; RRFS = Regional Recurrence Free Survival; MSM = Melanoma Specific Mortality; SEER = Surveillance, Epidemiology, and End Results Program; PLG = University of Pennsylvania's Pigmented Lesion Group; SLNB = Sentinel Lymph Node Biopsy; SNB = Sentinel Node Biopsy

For peer review only

Supplemental Table 6: Model evaluation

Citation	Predictors in final model	Type of model	Model performance			Validation method
			Discrimination	Calibration	Overall Performance	
Baade 2015[21]	Age, sex, smooth-rank transform thickness, body site, ulceration, positive lymph nodes, metastasis	Melanoma Severity Index	D-statistic = 1.50 (1.44 to 1.56) Harrell-C statistic = 0.88 (0.88 to 0.89)	NA	RD2 statistic = 0.47 (0.45 to 0.49)	Internal-external cross validation
Balch 2001[22]	Age, sex, ulceration, tumour thickness, anatomic level, nodal status, number of metastatic nodes, site	AJCC Validation	NA	NA	NA	External (geographical)
Cochran 2000a[23]	Age, sex, ulceration, site, Breslow thickness	OS Risk Score	NA	NA	NA	Internal (random split)
Cochran 2000b[23]	Ulceration, site, Breslow thickness, melanoma subtype	Recurrence Risk Score	NA	NA	NA	Internal (random split)
El Sharouni 2021a[24]	Age, ulceration, site, Breslow thickness, melanoma subtype, mitosis	LRFS Nomogram	C-statistic DEV: 0.79 (0.76 to 0.82) VALID: 0.80 (0.69 to 0.90)	Calibration plot	NA	Internal (bootstrap) + external (geographical)
El Sharouni 2021b[24]	Sex, ulceration, site, Breslow depth, melanoma subtype, SLN status, mitosis	RRFS Nomogram	C-statistic DEV: 0.77 (0.75 to 0.79) VALID: 0.76 (0.70 to 0.82)	Calibration plot	NA	Internal (bootstrap) + external (geographical)
El Sharouni 2021c[24]	Sex, ulceration, site, Breslow depth, SLN status, mitosis	DRFS Nomogram	C-statistic DEV: 0.80 (0.77 to 0.83) VALID: 0.74 (0.69 to 0.80)	Calibration plot	NA	Internal (bootstrap) + external (geographical)

For peer review only - http://bmjopen.bmj.com/site/about/guidelines.xhtml

Citation	Predictors in final model	Type of model	Model performance			Validation method
			Discrimination	Calibration	Overall Performance	
Fonseca 2020a[25]	Age, ulceration, Breslow thickness, mitotic rate, SLN status	DRFS Nomogram	Harrell C-index = 0.734	Calibration plot	NA	Internal (bootstrap)
Fonseca 2020b[25]	Ulceration, Breslow thickness, mitotic rate, SLN status, satellitosis	MSS Nomogram	Harrell C-index = 0.718	Calibration plot	NA	Internal (bootstrap)
Gimotty 2004[27]	Sex, growth phase (vertical or radial), vertical growth phase (VGP), VGP tumour-infiltrating lymphocyte	Metastasis Prognostic classification tree	Risk groups: AUC = 0.85<br>AJCC Stage: AUC = 0.59	NA	NA	External (new cohort)
Gimotty 2007a[26]	Sex, body site, ulceration, tumour thickness, invasion level	OS Prognostic classification tree	SEER: AUC = 0.76	NA	NA	External (geographical)
Gimotty 2007b[26]	Age, sex, clark level, vertical growth mitogenicity	OS Prognostic classification tree	PLG: AUC = 0.83	NA	NA	External (geographical)
Maurichi 2014[28]	Age, ulceration, mitotic rate, lymphovascular invasion, regression, sentinel node status	OS Nomogram	Harrell C statistic = 0.88	Calibration plot	NA	Internal
Rosenbaum 2017[29]	Histopathological width, conformation status (contiguous or noncontiguous), digital or manual area	RFS Nomogram	Youden Index of the AU-ROC curve: AUC = 0.733 (0.647 to 0.818)	NA	NA	Internal (ten-fold cross-validation)
Soong 2010[30]	Age, ulceration, tumour thickness, site, invasion level, lesion site	MSS nomogram<br><br>Web-based tool/ melanomaprognosis.org	NA	Correlation coefficients:<br>5 years = 0.90<br>10 years = 0.93	NA	External (geographical)

For peer review only - http://bmjopen.bmj.com/site/about/guidelines.xhtml

Citation	Predictors in final model	Type of model	Model performance			Validation method
			**Discrimination**	**Calibration**	**Overall Performance**	
Tsai 2007[31]	Age, sex, ulceration, tumour thickness, site, invasion level	Prognostic classification tree	NA	NA	Brier score<br>At 1 year: 0.02<br>At 15 years: 0.20	Internal (cross-validation)
Verver 2019a[32]	Ulceration, anatomical location and Breslow thickness	Recurrence Nomogram	C-index = 0.74	Calibration slope 1.10 [0.96 to 1.24]	NA	Internal-external cross validation
Verver 2019b[32]	Ulceration, anatomical location and Breslow thickness	MSM Nomogram	C-index = 0.76		NA	Internal-external cross validation
Vollmer 2001[33]	Age, sex, ulceration, tumour thickness, site	Scoring system	NA	NA	NA	Internal (two-fold cross-validation)
Xiao 2020[34]	Age, sex, race, marital status, anatomic site, stage, depth, ulceration, mitoses, treatment	OS Nomogram	C-index<br>DEV: 0.817 (0.811 to 0.823)<br>VALID: 0.817 (0.809 to 0.825)	Calibration plot	NA	Internal (split sample)

Abbreviations: MSS = Melanoma Specific Survival; OS = Overall Survival; DRFS = Distant Recurrence-Free survival; RFS = Recurrence Free Survival; RRFS = Regional Recurrence Free Survival; AU-ROC = Area under the Receiver Operating Characteristic

For peer review only

For peer review only - http://bmjopen.bmj.com/site/about/guidelines.xhtml

Supplemental Table 7: Risk of bias assessment

Study	Endpoint	ROB				Overall R.O.B
		Participants	Predictors	Outcome	Analysis	
Baade 2015[21]	MSS	+	?	+	?	?
Balch 2001[22]	MSS	+	+	+	?	?
Cochran 2000a[23]	OS	-	?	+	-	-
Cochran 2000b[23]	Recurrence	-	?	+	-	-
El Sharouni 2021a[24]	LRFS	+	?	+	+	?
El Sharouni 2021b[24]	RRFS	+	?	+	+	?
El Sharouni 2021c[24]	DRFS	+	?	+	+	?
Fonseca 2020a[25]	DFRS	+	?	+	-	-
Fonseca 2020b[25]	MSS	+	?	+	-	-
Gimotty et al. 2004[27]	Metastasis	+	?	+	+	?
Gimotty 2007a[26]	OS	+	+	+	?	?
Gimotty 2007b[26]	OS	+	+	+	?	?
Maurichi 2014[28]	OS	+	+	+	+	+
Rosenbaum 2017[29]	RFS	+	?	+	-	-
Soong 2010[30]	OS	+	+	+	+	+
Tsai 2007[31]	OS	+	+	+	+	+
Verver 2019a[32]	Recurrence	+	?	+	-	-
Verver 2019b[32]	MSM	+	?	+	-	-
Vollmer 2001[33]	MSM	+	+	+	?	?
Xiao 2020[34]	OS	+	?	+	-	-
+ Indicates low ROB/ low concern regarding applicability						
- Indicates high ROB/ high concern regarding applicability						
? Indicates unclear ROB/ unclear concern regarding applicability						

Abbreviations: ROB = Risk of Bias; MSS = Melanoma Specific Survival; OS = Overall Survival; DRFS = Distant Recurrence-Free survival; RFS = Recurrence Free Survival; RRFS = Regional Recurrence Free Survival; MSM = Melanoma Specific Mortality

For peer review only - http://bmjopen.bmj.com/site/about/guidelines.xhtml

Supplemental Table 8: Model Applicability Assessment

Study	Endpoint	Applicability			Overall Applicability
		Participants	Predictors	Outcome	
Baade 2015[21]	MSS	+	+	+	+
Balch 2001[22]	MSS	+	+	+	+
Cochran 2000a[23]	OS	-	+	+	-
Cochran 2000b[23]	Recurrence	-	+	+	-
El Sharouni 2021a[24]	LRFS	+	+	+	+
El Sharouni 2021b[24]	RRFS	+	+	+	+
El Sharouni 2021c[24]	DRFS	+	+	+	+
Fonseca 2020a[25]	DFRS	+	+	+	+
Fonseca 2020b[25]	MSS	+	+	+	+
Gimotty et al. 2004[27]	Metastasis	+	+	+	+
Gimotty 2007a[26]	OS	+	+	+	+
Gimotty 2007b[26]	OS	+	+	+	+
Maurichi 2014[28]	OS	+	+	+	+
Rosenbaum 2017[29]	RFS	+	+	+	+
Soong 2010[30]	OS	+	+	+	+
Tsai 2007[31]	OS	+	+	+	+
Verver 2019a[32]	Recurrence	+	+	+	+
Verver 2019b[32]	MSM	+	+	+	+
Vollmer 2001[33]	MSM	+	+	+	+
Xiao 2020[34]	OS	+	+	+	+
+ Indicates low ROB/ low concern regarding applicability					
- Indicates high ROB/ high concern regarding applicability					
? Indicates unclear ROB/ unclear concern regarding applicability					

Abbreviations: ROB = Risk of Bias; MSS = Melanoma Specific Survival; OS = Overall Survival; DRFS = Distant Recurrence-Free survival; RFS = Recurrence Free Survival; RRFS = Regional Recurrence Free Survival; MSM = Melanoma Specific Mortality

For peer review only - http://bmjopen.bmj.com/site/about/guidelines.xhtml

Supplemental File 1: Risk of bias assessment

Assessments were conducted using the PROBAST tool. Overall, eight models were judged to be at high risk of bias, three models were rated as low, and nine models were rated as having an unclear risk. Bias was introduced by various methods.

Selection of participants

Only two models, reported in one study,[23] were judged to be at high risk of bias. Very little detail was given on the participants in the study and there was no summary of patient characteristics to judge the severity of their melanoma. The rest of the studies were all classified as at low risk of bias.

Risk of bias introduced by predictors

Thirteen models (three in one study,[24] two each in three studies,[23,25,32] and four in four studies[21,27,29,34]), were rated to have an unclear risk of bias for selection of risk factors included in the final models. The models did not report information on blinding, and the predictors for these models were decided through backward variable selection procedure. With this method, variables are chosen based on significance level, meaning that variables regarded as insignificant, following univariate analysis, are removed from the model. However, the remaining seven models, reported in six studies,[22,26,28,30,31,33] were rated to have a low risk of bias for this domain. Although the model studies did not report information on blinding of predictor assessment to outcome data, their measurements can be inferred to be blinded to the outcome as predictors appeared to be measured and reported before the outcome occurred.

Risk of bias introduced by outcomes

All models had low concern regarding the timing of assessment. It was felt that the length of follow-up times for the outcomes assessed would not bias the prediction models.

Risk of bias introduced by the analysis

Eight models reported in five studies (two each in three studies,[23,25,32] and two in two studies[29,34]) were judged to be of high risk of bias. This was mainly due to the inclusion of variables identified as significant following a univariate analysis.[23,29,34] This method can lead to incorrect predictor selection because predictors are chosen on the basis of their statistical significance as a single predictor rather than in context with other predictors.[36,37] Dichotomization of continuous predictors was a concern for

For peer review only - http://bmjopen.bmj.com/site/about/guidelines.xhtml

some of the models.[25,34] This can potentially reduce a model's predictive ability, by assuming a different risk for different groups.[36,37] Relevant information on whether model overfitting and optimism in model performance was accounted for was not reported in one model.[32]

Concerns regarding applicability

Overall, only two models, reported in one study,[23] were rated as having a high-risk regarding applicability, and the remaining nineteen models were rated as having a low risk regarding applicability.

Concern regarding selection of participants

Only two models reported in one study,[23] were judged to have a high-risk of bias regarding selection of participants. Patients recruited for model development were a subset of the John Wayne Cancer Institute Melanoma database, however not much information was given regarding the characteristics of the patients or the severity of their melanoma. The rest of the models were rated as having a low concern because participant data were taken from routine care or cancer registries, the participants included in the studies matched the participants in the review question. Therefore, the characteristics of patients in the study were likely to match patients with similar characteristics presenting in either a different setting or country.

Concern regarding assessment and timing of predictors

All models had low concern regarding the definition, assessment, and timing of the predictors. All predictors were measured using methods potentially applicable to the daily practice that is addressed by the review.

Concern regarding the applicability of the outcome determined

All models were judged to have a low concern regarding the applicability of the outcomes. Our systematic review explicitly aimed to include all validations of the model regardless of outcome definition and measurement method.

For peer review only

For peer review only - http://bmjopen.bmj.com/site/about/guidelines.xhtml

PRISMA Checklist

Section/topic	#	Checklist item	Reported on page #
**TITLE**			
Title	1	Identify the report as a systematic review, meta-analysis, or both.	1
**ABSTRACT**			
Structured summary	2	Provide a structured summary including, as applicable: background; objectives; data sources; study eligibility criteria, participants, and interventions; study appraisal and synthesis methods; results; limitations; conclusions and implications of key findings; systematic review registration number.	3
**INTRODUCTION**			
Rationale	3	Describe the rationale for the review in the context of what is already known.	4
Objectives	4	Provide an explicit statement of questions being addressed with reference to participants, interventions, comparisons, outcomes, and study design (PICOS).	4
**METHODS**			
Protocol and registration	5	Indicate if a review protocol exists, if and where it can be accessed (e.g., Web address), and, if available, provide registration information including registration number.	5
Eligibility criteria	6	Specify study characteristics (e.g., PICOS, length of follow-up) and report characteristics (e.g., years considered, language, publication status) used as criteria for eligibility, giving rationale.	5
Information sources	7	Describe all information sources (e.g., databases with dates of coverage, contact with study authors to identify additional studies) in the search and date last searched.	5
Search	8	Present full electronic search strategy for at least one database, including any limits used, such that it could be repeated.	5
Study selection	9	State the process for selecting studies (i.e., screening, eligibility, included in systematic review, and, if applicable, included in the meta-analysis).	5
Data collection process	10	Describe method of data extraction from reports (e.g., piloted forms, independently, in duplicate) and any processes for obtaining and confirming data from investigators.	5
Data items	11	List and define all variables for which data were sought (e.g., PICOS, funding sources) and any assumptions and simplifications made.	5
Risk of bias in individual studies	12	Describe methods used for assessing risk of bias of individual studies (including specification of whether this was done at the study or outcome level), and how this information is to be used in any data synthesis.	5
Summary measures	13	State the principal summary measures (e.g., risk ratio, difference in means).	5
Synthesis of results	14	Describe the methods of handling data and combining results of studies, if done, including measures of consistency (e.g., I^2) for each meta-analysis.	5

For peer review only - http://bmjopen.bmj.com/site/about/guidelines.xhtml

PRISMA Checklist

Section/topic	#	Checklist item	Reported on page #
Risk of bias across studies	15	Specify any assessment of risk of bias that may affect the cumulative evidence (e.g., publication bias, selective reporting within studies).	N/A
Additional analyses	16	Describe methods of additional analyses (e.g., sensitivity or subgroup analyses, meta-regression), if done, indicating which were pre-specified.	N/A
**RESULTS**			
Study selection	17	Give numbers of studies screened, assessed for eligibility, and included in the review, with reasons for exclusions at each stage, ideally with a flow diagram.	6
Study characteristics	18	For each study, present characteristics for which data were extracted (e.g., study size, PICOS, follow-up period) and provide the citations.	6
Risk of bias within studies	19	Present data on risk of bias of each study and, if available, any outcome level assessment (see item 12).	9
Results of individual studies	20	For all outcomes considered (benefits or harms), present, for each study: (a) simple summary data for each intervention group (b) effect estimates and confidence intervals, ideally with a forest plot.	7-8
Synthesis of results	21	Present results of each meta-analysis done, including confidence intervals and measures of consistency.	N/A
Risk of bias across studies	22	Present results of any assessment of risk of bias across studies (see Item 15).	N/A
Additional analysis	23	Give results of additional analyses, if done (e.g., sensitivity or subgroup analyses, meta-regression [see Item 16]).	N/A
**DISCUSSION**			
Summary of evidence	24	Summarize the main findings including the strength of evidence for each main outcome; consider their relevance to key groups (e.g., healthcare providers, users, and policy makers).	9-12
Limitations	25	Discuss limitations at study and outcome level (e.g., risk of bias), and at review-level (e.g., incomplete retrieval of identified research, reporting bias).	11
Conclusions	26	Provide a general interpretation of the results in the context of other evidence, and implications for future research.	12
**FUNDING**			
Funding	27	Describe sources of funding for the systematic review and other support (e.g., supply of data); role of funders for the systematic review.	1

From: Moher D, Liberati A, Tetzlaff J, Altman DG, The PRISMA Group (2009). Preferred Reporting Items for Systematic Reviews and Meta-Analyses: The PRISMA Statement. PLoS Med 6(7): e1000097. doi:10.1371/journal.pmed1000097

For more information, visit: **www.prisma-statement.org**.

For peer review only - http://bmjopen.bmj.com/site/about/guidelines.xhtml

BMJ Open

Predictive Accuracy of Risk Prediction Models for Recurrence, Metastasis and Survival for Early-Stage Cutaneous Melanoma: A Systematic Review

Journal:	*BMJ Open*
Manuscript ID	bmjopen-2023-073306.R1
Article Type:	Original research
Date Submitted by the Author:	25-Aug-2023

Complete List of Authors:	Kunonga, Tafadzwa; Newcastle University, Evidence Synthesis Group, Population & Health Sciences Institute; Newcastle University, NIHR Innovation Observatory, Population Health Sciences Institute<br>Kenny, RPW; Newcastle University, Evidence Synthesis Group, Population & Health Sciences Institute; Newcastle University, NIHR Innovation Observatory, Population Health Sciences Institute<br>Astin, Margaret; Newcastle University, Evidence Synthesis Group, Population & Health Sciences Institute<br>Bryant, Andrew; Newcastle University, Biostatistics Research Group, Population Health Sciences Institute<br>Kontogiannis, Vasileios; Newcastle University, Health Economics Group, Population Health Sciences Institute<br>Coughlan, Diarmuid; Newcastle University, Health Economics Group, Population Health Sciences Institute<br>Richmond, Catherine; Newcastle University, Evidence Synthesis Group, Population & Health Sciences Institute; Newcastle University, NIHR Innovation Observatory, Population Health Sciences Institute<br>Eastaugh, Claire H.; Newcastle University, Evidence Synthesis Group, Population & Health Sciences Institute; Newcastle University, NIHR Innovation Observatory, Population Health Sciences Institute<br>Beyer, Fiona ; Newcastle University, Evidence Synthesis Group, Population & Health Sciences Institute; Newcastle University, NIHR Innovation Observatory, Population Health Sciences Institute<br>Pearson, F; Newcastle University, Evidence Synthesis Group, Population & Health Sciences Institute; Newcastle University, NIHR Innovation Observatory, Population Health Sciences Institute<br>Craig, Dawn; Newcastle University, Evidence Synthesis Group, Population & Health Sciences Institute; Newcastle University, NIHR Innovation Observatory, Population Health Sciences Institute<br>Lovat, Penny; Newcastle University, Dermatological Sciences, Translation and Clinical Research Institute; AMLo Biosciences, The Biosphere, Newcastle Helix, Newcastle upon Tyne, NE4 5BX<br>Vale, Luke; Newcastle University, Health Economics Group, Population Health Sciences Institute<br>Ellis, Robert; Newcastle University, Dermatological Sciences, Translational Research Institute; South Tees Hospitals NHS FT, Department of Dermatology

Primary Subject	Oncology

Heading:	
Secondary Subject Heading:	Dermatology
Keywords:	Systematic Review, Adult oncology < ONCOLOGY, Dermatological tumours < ONCOLOGY, Epidemiology < ONCOLOGY

SCHOLARONE™
Manuscripts

I, the Submitting Author has the right to grant and does grant on behalf of all authors of the Work (as defined in the below author licence), an exclusive licence and/or a non-exclusive licence for contributions from authors who are: i) UK Crown employees; ii) where BMJ has agreed a CC-BY licence shall apply, and/or iii) in accordance with the terms applicable for US Federal Government officers or employees acting as part of their official duties; on a worldwide, perpetual, irrevocable, royalty-free basis to BMJ Publishing Group Ltd ("BMJ") its licensees and where the relevant Journal is co-owned by BMJ to the co-owners of the Journal, to publish the Work in this journal and any other BMJ products and to exploit all rights, as set out in our licence.

The Submitting Author accepts and understands that any supply made under these terms is made by BMJ to the Submitting Author unless you are acting as an employee on behalf of your employer or a postgraduate student of an affiliated institution which is paying any applicable article publishing charge ("APC") for Open Access articles. Where the Submitting Author wishes to make the Work available on an Open Access basis (and intends to pay the relevant APC), the terms of reuse of such Open Access shall be governed by a Creative Commons licence – details of these licences and which Creative Commons licence will apply to this Work are set out in our licence referred to above.

Other than as permitted in any relevant BMJ Author's Self Archiving Policies, I confirm this Work has not been accepted for publication elsewhere, is not being considered for publication elsewhere and does not duplicate material already published. I confirm all authors consent to publication of this Work and authorise the granting of this licence.

For peer review only

Predictive Accuracy of Risk Prediction Models for Recurrence, Metastasis and Survival for Early-Stage Cutaneous Melanoma: A Systematic Review

Authors

Tafadzwa Patience Kunonga,[1,2] Ryan P.W. Kenny,[1,2] Margaret Astin[1] Andy Bryant,[3] Vasileios Kontogiannis,[4] Diarmuid Coughlan,[4] Catherine Richmond,[1,2] Claire H. Eastaugh,[1,2] Fiona R. Beyer,[1,2] Fiona Pearson,[1,2] Dawn Craig,[1,2,4] Penny Lovat,[5,6] Luke Vale,[4] Robert Ellis,[5,6,7]

1. Evidence Synthesis Group, Population Health Sciences Institute, Newcastle University
2. NIHR Innovation Observatory, Population Health Sciences Institute, Newcastle University
3. Biostatistics Research Group, Population Health Sciences Institute, Newcastle University
4. Health Economics Group, Population Health Sciences Institute, Newcastle University
5. Dermatological Sciences, Translation and Clinical Research Institute, Newcastle University
6. AMLO Biosciences, The Biosphere, Newcastle Helix, Newcastle upon Tyne, NE4 5BX
7. Department of Dermatology, South Tees Hospitals NHS FT

Corresponding author:

Tafadzwa Patience Kunonga, Evidence Synthesis Group, Population Health Sciences Institute, Baddiley Clark Building, Newcastle University, Newcastle upon Tyne NE2 4BN, patience.kunonga@newcastle.ac.uk

Funding/support:

This work was supported by the National Institute for Health Research (NIHR) Health Technology Assessment Programme, grant number 16/166/05 and the NIHR Invention for Innovation (i4i) Innovative Prognostic Test for Early-Stage Cutaneous Melanoma, grant number 20993.

Role of the Funder/Sponsor:

The funding sources had no role in the design and conduct of the study; collection, management, analysis, and interpretation of the data; preparation, review, or approval of the manuscript; and the decision to submit the manuscript for publication.

For peer review only - http://bmjopen.bmj.com/site/about/guidelines.xhtml

Conflict of Interest Disclosures:

Kunonga, Kenny, Astin, Bryant, Kontogiannis, Coughlan, Richmond, Eastaugh, Beyer, Pearson, Nasr have no conflicts of interest to declare. Craig - December 2018 to present, member of the HD&DR Research-led prioritisation committee. Vale was a Member of the NIHR Clinical Evaluation and Trial Panel from 2015-2018. Vale and Craig are both part funded by the NIHR Applied Research Collaboration for the North East and North Cumbria. Lovat is Chief Scientific Officer for AMLo Biosciences Ltd, holds shares in AMLo and is named as an inventor on patents for biomarkers this area. Ellis received personal fees from AMLo Biosciences Ltd, outside the submitted work, he also was previously Chief Medical Officer for AMLo Biosciences Ltd but gave up this position in January 2021. He is named as an inventor on patents owned by AMLo Biosciences Ltd for biomarkers this area.

Data availability statement

All data relevant to the study are included in the article or uploaded as supplemental information.

Word count

Abstract: 294

Manuscript: 3, 846

Tables: 3

Figures: 1

For peer review only

For peer review only - http://bmjopen.bmj.com/site/about/guidelines.xhtml

Abstract

Objectives

To identify prognostic models for melanoma survival, recurrence, and metastasis among AJCC stage I and II patients post-surgery; and evaluate model performance, including overall survival prediction.

Design

Systematic review and narrative synthesis.

Data Sources

Searched MEDLINE, Embase, CINAHL, Cochrane Library, Science Citation Index, and grey literature sources including cancer and guideline websites from 2000 to September 2021.

Eligibility Criteria

Included studies on risk prediction models for stage I and II melanoma in adults ≥18 years. Outcomes included overall survival, recurrence, metastases, and model performance. No language or country of publication restrictions were applied.

Data Extraction and synthesis

Two pairs of reviewers independently screened studies, extracted data, and assessed the risk of boas using the CHecklist for critical Appraisal and data extraction for systematic Reviews of prediction Modelling Studies checklist and the Prediction study Risk of Bias Assessment Tool. Heterogeneous predictors prevented statistical synthesis.

Results

From 28,967 records, fifteen studies reporting twenty models were included; eight (stage I), two (stage II), seven (stages I-II), and three (stages not reported), but were clearly applicable to early stages. Clinicopathologic predictors per model ranged from three to ten. The most common were: ulceration, Breslow thickness/depth, sociodemographic status, and site. Where reported, discriminatory values were ≥ 0.7. Calibration measures showed good matches between predicted and observed rates. None of the studies assessed clinical usefulness of the models. Risk of bias was high in eight models, unclear in nine and low in three. Seven models were internally and externally cross-validated, six models were externally validated, and eight models were internally validated.

For peer review only

Conclusions

All models are effective in their predictive performance, however the low quality of the evidence raises concern as to whether current follow-up recommendations following surgical treatment is adequate. Future models should incorporate biomarkers for improved accuracy.

Key words: early-stage melanoma, prediction model, systematic review, recurrence, survival, metastasis

Article Summary

Strengths and limitations of this study

- Comprehensive and systematic searches captured the core evidence about prediction models in early-stage melanoma.
- The current evidence reviewed across all models is insufficient to make recommendations regarding use in clinical practice.
- Heterogeneity in the data across study populations, predictors, and clinical progression of the disease suggests there is insufficient evidence to make firm conclusions about best clinical practice in the application of predictive models in patients with AJCC stage I or II cutaneous melanoma.

For peer review only - http://bmjopen.bmj.com/site/about/guidelines.xhtml

Introduction

Melanoma is one of the deadliest of all skin cancers.[1] The worldwide incidence of melanoma is estimated to be approximately 2% of cancers per annum.[2] However, in early-stage disease,, once surgically removed from the skin, through wide local excision (WLE), and without adjuvant immunotherapy, chemotherapy or radiotherapy, the majority of early-stage melanomas are cured, with an estimated 5 –year overall survival rate of 95%-100% (stage I), and 65%-92.8% (stage II).[3] However, up to 30% of all primary melanomas progress to metastatic disease with an associated extremely poor survival rate of only 5 – 15%.[4] Although the surgical treatment of primary melanoma is effective and long established, there has been a rapid pace of change recently with the addition of sentinel lymph node biopsy (SLNB).[5] This procedure identifies the sentinel lymph node which is excised for examination to determine whether cancer cells are present.[6]

Currently, a structured, uniformly adopted, evidence-based model of patient follow-up after initial diagnosis and treatment is lacking.[7] Follow-up strategies depend on the stage and progression of the disease; based on the criteria defined within the 8th edition of the American Joint Committee on Cancer (AJCC) staging manual.[8] For this review, however, the definitions of stage and progression used in the 2008, 7th edition[9] and earlier editions remain relevant as the reported data in research studies may be based on definitions from earlier editions. Current guidelines for management of the condition vary across the world, with most developed using anecdotal evidence and expert opinion. A clinical prediction model, a statistical tool that relates multiple factors to the probability of a patient having a future clinical event,[10] could be used to aid early detection of disease progression such as new primary tumours, in-transit metastasis, or locoregional recurrence in the regional lymph nodes. Before introducing a new clinical prediction model in to practice there needs to be evidence of model development, validation and impact. Validation should involve evaluating performance (in terms of discrimination or calibration), and clinical usefulness in practice. Impact should consider whether the model improves decision-making.[10]

Previous systematic reviews have been conducted looking at the value of risk prediction models for melanoma development.,[11-13] and clinical risk of survival of melanoma.[14] However, to our knowledge, no review has critically appraised current clinicopathologic prediction models for primary melanomas (stages I and II), following surgical excision of the tumour. This review aims to identify

For peer review only - http://bmjopen.bmj.com/site/about/guidelines.xhtml

and assess prognostic/predictive models used to predict patient survival, recurrence (any site), and metastasis in AJCC stage (I and II) melanoma following surgical excision, thus potentially allowing further refinement of risk-stratification of patients.

Methods

The study protocol is published on PROSPERO (CRD42018086784). The review population listed in the protocol was later augmented to include stage II melanoma. As with stage I melanoma, tumours at this stage may not have advanced and are treated with WLE.[15] The review followed the Preferred Reporting Items for Systematic Reviews and Meta-Analyses guideline (PRISMA).[16]

Search strategy

Searches were originally conducted in July 2019, then updated in September 2021. A date restriction of 2000 was utilised, as this is when SLNB became routine in the USA.[5] The search strategy (Supplemental Table 1 and Supplemental Table 2) was designed in MEDLINE and translated to other databases (Supplemental Table 3). A published and validated prognostic study filter[17] was used including the following concepts: [melanoma] AND [risk models] AND [prognosis]. No language or country of publication restrictions were applied. In the search update (June 2019 to September 2021), a more up to date search filter was used.[18]

Inclusion and exclusion criteria

Studies were selected using piloted inclusion criteria (Table 1) according to the Patient population, Intervention, Comparator, Outcome, Timing and Setting (PICOTS) formula.[19] The primary outcome is overall survival (OS) defined as patient survival until death from melanoma following primary treatment. Secondary outcomes include: number of detected recurrence and metastasis, and the discrimination, calibration, overall performance, and clinical utility of the models. A table of definitions for the performance measures is presented in Supplemental Table 4.

Selection of studies

Two reviewers independently screened titles and abstracts for articles relevant to stage I disease. For stage II screening a text mining approach was adopted, followed by duplicate and independent hand screening of study abstracts. Two reviewers independently screened the selected full text articles. For each included study, data were extracted by one of four reviewers using the CHecklist for critical

For peer review only - http://bmjopen.bmj.com/site/about/guidelines.xhtml

Appraisal and data extraction for systematic Reviews of prediction Modelling Studies (CHARMS) checklist.[20] Data were extracted for characteristics of participants, outcomes, predictors, model development methods, model performance and validation. Extraction forms were checked for accuracy and consistency by another reviewer, and a clinical expert.

Risk of bias assessment

Two pairs of reviewers used the Prediction study Risk of Bias Assessment Tool (PROBAST),[21] to assess the risk of bias (ROB) of each study.. The tool, which has gained popularity on reviews of prognostic studies,[22] evaluates 20 questions in four domains (Participants, Predictors, Outcome, and Analysis) to assess the ROB and applicability of prediction model studies.[21] Following the guide, we used the signalling questions across these domains to make the following judgements: “yes” means low bias, “no” means high bias. For the overall judgement, the study was rated as low ROB if all domains were rated as low, high ROB if at least one domain was rated as high. Where there was insufficient information to make a judgement, we rated the domain as unclear ROB. However, if one domain was rated as unclear and the others low, overall judgement was rated as unclear..[21]

Data analysis and synthesis

A narrative synthesis was undertaken, including summarising the characteristics of the included models and the performance of prediction models, focusing on measures of discrimination, calibration, and overall performance; and model validation methods. We were unable to conduct a meta-analysis due to the variance in use of disparate predictors, and modelling methods. Furthermore, it was not possible to perform subgroup analyses as the studies identified did not report outcomes by population subgroups.

Quality of the evidence

We had planned to assess the overall quality of evidence using the Grading of Recommendations Assessment, Development and Evaluation (GRADE).[23] However, although the tool has been adapted for assessing overall quality in prognostic factor reviews, studies, it is yet to be adapted for prediction model reviews.[24]

Insert Table 1 here

For peer review only - http://bmjopen.bmj.com/site/about/guidelines.xhtml

Patient and Public Involvement

This review was requested by our funder within a timescale that did not allow for meaningful public and patient involvement.

Results

Selection of studies

We identified 28,967 records after deduplication, of which 165 were selected for full text screening (Supplemental Figure 1). Fourteen studies reporting twenty unique risk prediction models, met the inclusion criteria.[25-38]

Characteristics of studies

Characteristics of included studies are presented in Table 2. Studies were published between 2000 and 2021; nine from the USA,[26 27 30 31 33-35 37 38] two from Australia,[25 28] two from Europe,[32 36] and one from Brazil.[29] Twelve studies were from retrospective cohorts,[25-32 36-38] and three were from prospective cohorts.[33-35] Patient data were obtained from population-based cancer registries, medical/clinical records or a combination of these sources. Study intervals ranged from 1972 to 2015. Eight models were developed from patients with stage I melanoma only, (three from one study,[28] two from another study,[30] and three from three studies[31-33]), two for stage II only (both reported in the same study[29]), eight for stages I-II (two reported in one study,[36] and five from five studies[25 26 34 35 38]), and for three models the stages not reported (two from one study,[27] and the other from one study[37]), however they were found to be applicable to stages I-II. Eleven studies used logistic regression methods to develop the models,[25-29 32-34 36-38] and three used a recursive partitioning method.[30 31 35] Duration of follow-up ranged from three[38] to twenty years.[26]

Insert Table 2 here

Characteristics of included models

Characteristics of included models are presented in Table 3. Model outcomes included OS;[25 27 30 32 34 35 38] melanoma specific survival (MSS);[25 26 29] melanoma specific mortality (MSM);[36] mortality;[37] recurrence;[27 36] recurrence free survival (RFS);[33] local RFS (LRFS);[28] regional

For peer review only - http://bmjopen.bmj.com/site/about/guidelines.xhtml

RFS (RRFS)[28]; distant RFS (DRFS);[28 29] and metastasis.[31] A table of definitions used in the studies for model outcomes is presented in Supplemental Table 5. Total sample sizes across the models ranged from 259,[29] to 66,192 patients.[38] Five studies selected candidate clinico-pathologic variables based on previous clinical knowledge or literature (pre-specification),[25 28 30 35 37] three conducted a multivariate analyses,[30 32 34] two studies conducted a univariate analysis,[29 33] while three employed a combination of previous knowledge and univariate analysis.[27 36 38] One study did not provide information on variable selection.[26] A backward elimination analysis approach was used in seven studies,[25 27-29 31 33 36] and a full model approach was used in six studies,[30 32 34 35 37 38] to select the final predictors for model development. The number of predictors in the final models ranged from three[33 36] to ten.[38] The most common predictors were ulceration (17/20), Breslow thickness/depth (16/20), sociodemographic status (15/20), and body/anatomic site (14/20) (See Figure 1). The models are reported based on first authors and outcome as follows: Baade MSS model;[25] , Balch MSS model;[26] Cochran OS;[27] Cochran recurrence risk score;[27] El Sharouni LRFS nomogram;[28] El Sharouni, RRFS nomogram;[28] El Sharouni DRFS nomogram;[28] Fonseca,DRFS nomogram;[29] Fonseca MSS nomogram;[29] Gimotty metastasis nomogram;[31] Gimotty(a) OS model;[30] Gimotty(b) OS model;[30] Maurichi OS model;[32] Rosenbaum RFS model;[33] Soong OS model;[34] Tsai OS model;[35] Verver MSM model;[36] Vollmer mortality model;[37] and Xiao OS model.[38]

Insert Table 3 here

Performance of prediction models

Discrimination

The AUC-ROC was reported in two models (reported in one study) predicting OS,[30] one model predicting RFS,[33] and another predicting metastasis outcomes,[31] all with values above 0.7. The C-index measure was reported in the Maurichi OS model;[32] Xiao OS model;[38] El Sharouni DRFS model;[28] Fonseca DRFS model;[29] El Sharouni LRFS model;[28] El Sharouni RRFS model;[28] Fonseca MSS model;[29] and Verver MSM model,[36]

all with values above 0.7 for the models. The discriminative performance of the models is considered acceptable when the AUC-ROC statistics and their equivalent are ≥ 0.7.[39] In addition to the C-index,

For peer review only - http://bmjopen.bmj.com/site/about/guidelines.xhtml

the Baade MSS model also estimated the D-statistic and reported a value of 1.50 (95% CI 1.44 to 1.56) for OS.[25]

Calibration

Calibration scores were reported in the Soong OS model,[34] with correlation coefficients of 0.90 and 0.93 for five- and ten-year survival rates.[34] Calibration plots for predicting survival outcomes were presented in ten models: Maurichi OS model;[32] Xiao OS model;[38] El Sharouni DRFS model;[28] Fonseca DRFS model;[29] El Sharouni RRFS model;[28] El Sharouni LRFS model;[28] Fonseca MSS model;[29] Verver recurrence model;[36] and Verver MSM model.[36] Three models that reported callibration scores reported values closer to 1, indicating a perfect agreement, while eight models graphically presented plots showing a good match between predicted and observed outcomes for OS, DRFS, RRFS, LRFS, MSS, and metastasis. This suggests that the models have the ability to accurately generate predictions that are close to the observed outcomes. Results indicated high accuracy and precision of the prediction models, as predicted and actual survival probabilities were similar.[40]

Overall Performance

The Baade OS model assessed the overall performance of predicting OS by assessing how well the model fit the data using the r-squared (R^2) statistic.[25] The model reported the R^2 as 0.47 (0.45 to 0.49), equating to a strong fit.[25] The Tsai OS model,[35] measured overall performace by assessing the Brier score, with usual values ranging from 0 (total accuracy) to 1 (wholly innacurate).[41] Results show that the score for the proposed, progonstic classification scheme increased with time from 0.02 at year one to approximately 0.20 at year 15, indicating high accuracy in predicting OS.[35]

Clinical utility

None of the studies conducted either a net benefit or decision curve analysis, therefore we were unable to address the clinical utility of the tools. The clinical utility of a validated risk prediction model would help clinicians and patients during the surveillance phase of care.[42]

For peer review only - http://bmjopen.bmj.com/site/about/guidelines.xhtml

Model validation methods

Model validation methods are presented in Supplemental Table 6. Internal validation refers to the performance of a model developed and tested on a sample of the same population.[43] External validation refers to how well a model predicts an outcome in a data set that is different to the development population (new cohort), or a different region or country (geographical).[44] Eight models were internally validated. The Rosenbaum RFS model,[33] and Vollmer mortality model,[37] used the k-fold cross-validation procedure, where an original sample is randomly partitioned into *k* equal-sized subsamples.[45] The Cochran OS model,[27] Cochran recurrence model,[27] and Xiao OS model,[38] randomly selected a proportion of the sample and retained these as the validation set, using the rest for training. The Maurichi OS model.[32] was validatedby assessing the congruence of expected outcomes (predicted from the model) and observed outcomes. The Fonseca DRFS model,[29] and Fonseca MSS model,[29]were internally validated using bootstrap methods. Bootstrapping entails repeatedly drawing random samples with replacement from the data to construct prediction models and evaluating model performance using out-of-bag observations..[41] Five models validated their models using external techniques, including geographic validation: the Gimotty(a) OS model;[30] Gimotty(b) OS model;[30] Balch MSS model;[26] Soong OS model;[34] and Gimotty metastasis model.[31]. Seven models: El Sharouni LRFS model;[28] El Sharouni RRFS model;[28] El Sharouni DRFS model;[28] Verver receurrence model;[36] Verver MSM model;[36] Baade MSS model,[25] and the Tsai OS model,[35], assessed model performance using internal-external cross-validation methods (IECV). IECV analyses the performance of models using new patients from different but related practices as compared to the original development sample.[41]

Critical appraisal of included studies

The results of the critical appraisal are presented in Supplemental Table 7. Overall, eight models: Cochran OS model;[27] Cochran recurrence model;[27] Fonseca DRFS model;[29] Fonseca MSS model;[29] Verver recurrence model;[36] Verver MSM model;[36] Rosenbaum RFS model;[33] and the Xiao OS model,[38] were judged to be at high risk of bias. Three models: Maurichi OS model;[32] Soong OS model;[34] and Xiao OS model,[38] were rated as low risk. Nine models: El Sharouni LRFS model;[28] El Sharouni RRFS model;[28] El Sharouni DRFS model;[28] Gimotty(a) OS model;[30] Gimotty(b) model;[30] Baade OS model;[25] Balch OS model;[26] Gimotty metastasis model;[30] and Vollmer mortality model,[37]. The most notable cause of high risk was for the analysis

For peer review only - http://bmjopen.bmj.com/site/about/guidelines.xhtml

domain. This was mainly due to the inclusion of variables in the final model, previously identified as significant following a univariate analysis.[27 29 33 38] This method can lead to incorrect predictor selection because predictors are chosen on the basis of their statistical significance as a single predictor rather than in context with other predictors.[41]

The applicability ratings of the models are presented in Supplemental Table 8. Two models: Cochran OS model,[27] and Cochran recurrence model, were rated as having a high risk regarding applicability, the rest of the models were rated as low risk of bias regarding applicability. Detailed information on critical appraisal is available in Supplemental File 1. The main concern regarding applicability was for the selection of participants in the study by Cochran et al.,[27] where little information is reported about the characteristics of the patients or the severity of their melanoma.

Discussion

This review identified 14 studies describing 20 different models developed for the prediction of recurrence, new primary tumours or metastasis in patients with AJCC stage I or II cutaneous melanoma following excision. The models differed in the predictors used depending on the outcome of interest and statistical measures used to assess model performance. It was therefore inappropriate to statistically synthesise their results. One of the limitations of the studies was the lack of reporting of baseline rates of SLNB, a technique useful for providing disease stage and to guide adjuvant systemic therapy.[46] The absence of these rates makes it difficult to be sure that the patients are correctly classified in the studies. As the AJCC classification criteria have changed over time, studies from different periods used different staging systems and methodologies. For models at stages I or II we, considered which AJCC staging criteria would apply taking into account reported factors such as tumour thickness, and ulceration (see Table 1). However, this can lead to significant heterogeneity in the data, making it difficult to compare and synthesise findings. Common risk factors included in the models were consistent with well-established risk factors for melanoma, including ulceration, age, sex, Breslow thickness, and tumour site.[47] Although systematic reviews of prediction models for cutaneous melanoma[11 12] have previously been conducted, to the best of our knowledge, this review investigates the potential of clinical models to predict recurrence, metastases, and survival of AJCC stage I and II melanoma following surgical excision. Similar to the reviews by Vuong et al.,[12]

For peer review only - http://bmjopen.bmj.com/site/about/guidelines.xhtml

and Mahar et al.,[14] notable differences in the approaches used to select predictors during model development were observed. Eight models selected predictors based on univariate analysis of the strength of these predictors, of which four were developed based on previous knowledge. When developing models, building on previous literature and expert clinician opinions is recommended. Building models based only on the statistical significance of the association between predictor and outcome in univariate analyses risks missing important predictors.[48]

Model performance measures were available for assessing discrimination in fourteen models, callibration in eleven models, and overall performance in two models. Whilst the type of discrimination statistic varied, the discrimination statistics of the new models ranged from 0.72 to 0.88, a range comparable to that reported in other published melanoma reviews: 0.62 to 0.86,[12] 0.7 to 0.8,[11] and 0.62 to 0.98.[14] Currently, there is a lack of evidence for the clinical performance of early stage melanoma prediction models based on clinicopathologic variables. Accuracy measures such as calibration and discrimination, however, do not provide any detail regarding a models suitability for clinical practice. Net benefit, obtained via decision curve analysis, aims to assess the clinical utility of such models.[42] Unfortunately, the studies included in this review do not give detail on clinical utility for the models they report upon. In addition, we reviewed follow up schedules as recommended by clinical guidelines and note the limited evidence base on which they are based.[49] This suggests more detailed studies about timing and frequency of relapse are needed. Most of the models were externally validated, either in new cohorts or patients from a different location or through IECV. External validation of models is essential to support the general applicability of any prediction model.[48] However, most of the models were rated as being at high risk of bias implying low confidence in the performance of these models in new datasets. This is because a high risk of bias can lead to either overestimation or underestimation of the predictive accuracy of a model, which can affect its generalisability to new datasets.[44]

This review followed procedures documented by the Cochrane Collaboration for conducting systematic reviews, the CHARMS[20] guidelines for extracting data, the PRISMA[16] guidelines for reporting and PROBAST tool[21] for assessing risk of bias, so was therefore robust. The update search conducted in September 2021 incorporated emerging trial evidence, so we are confident that

For peer review only - http://bmjopen.bmj.com/site/about/guidelines.xhtml

For peer review only

we would have identified relevant trials/studies coming to light in the next 1-2 years from the search implementation. Although the PROBAST tool provides a structured framework for evaluating various aspects of study methodology, like any other tool, it has its limitations. From our limited experience of the tool, we found that its effectiveness relied heavily on the accuracy and comprehensiveness of information available in the included studies. Most of the studies incorporated within our analysis were conducted before guidelines for conducting prognostic studies were developed, leading to missing or inconsistent information, thereby impeding a comprehensive application of the PROBAST tool to adequately appraise bias. A critical analysis of the PROBAST tool was critical of the inter-rater reliability (IRR) of the tool before training.[50] Although we did not calculate the IRR, we assessed RoB in two pairs to ensure consistent and reliable assessments. .

A recent guideline has been developed in 2015, the Transparent Reporting of a multivariable prediction model for Individual Prognosis or Diagnosis (TRIPOD), to ensure comprehensive reporting of critical methodological elements in individual prognostic studies.[51] Nevertheless, it is imperative to acknowledge that a limitation of the current review stems from the fact that the included studies were published prior to the availability of this reporting guidance. Recent evaluation of study quality based on the TRIPOD statement reveals insufficient reporting levels for risk prediction models in cutaneous melanoma. Embracing reporting guidelines such as TRIPOD, can enhance future research reporting standards.[52] Secondly, none of the studies reported a decision curve analysis to assess the clinical implications of the models. Thirdly, comparing the included models was problematic and meta-analysis was not feasible due to the variety in the predictors and statistical measures used for model performances. Lastly, we were unable to access the development and validation studies of some of the publicly available online tools, and these were excluded from our analysis.[53 54]

The results of this systematic review highlight the relative lack of appropriate evidence underpinning current melanoma prediction tools to support practice in AJCC stage I and II disease. The evidence needed for clinical guideline decision-makers to incorporate a prediction model into routine clinical care needs to be far more convincing than what has been reported thus far. The goal should be clear demonstration of external validity, by evaluating model transportability in other cohorts,[10] and clinical utility (the ability to better select patients from high risk groups for adjuvant therapy in the

For peer review only - http://bmjopen.bmj.com/site/about/guidelines.xhtml

future) by undertaking decision curve analysis to identify net benefits.[42 55] Additionally, biomarkers play a crucial role in risk prediction models of cutaneous melanoma.[56] These may include genetic, immune or circulatory biomarkers.[56] They are measurable characteristics that provide information about the disease's presence, progression, and response to treatment.[56] The relationship between biomarkers and clinico-pathological characteristics has been shown to be interconnected in predicting chemotherapy response in breast cancer.[57] Therefore, incorporating biomarkers into risk prediction models alongside clinico-pathological factors, may provide additional information to refine risk prediction models in cutaneous melanoma and aid healthcare professionals obtain more comprehensive and accurate assessments of an individual's risk. Additionally, other studies have shown that mutation status is associated with survival outcomes,[58 59] however we did not evaluate these studies as they were not developed into risk models. Critically, these results clearly outline the need for ongoing prognostication studies and, as such, this review acts as an evidence base to catalyse project development and funding, and future follow-up guidelines and management of patients, given the relative scarcity of evidence-based practice at present.

Conclusion

The data, which are heterogeneous in terms of biology and progression, do not offer a wide enough scope of best practice to allow accurate prognostication of melanoma AJCC stages I or II patients as defined by the criteria within the 3rd, 6th, 7th and 8th editions of AJCC staging, or recommendation for use in clinical practice. This raises concern as to whether current follow-up recommendations post-surgical treatment is adequate as the evidence supporting such recommendation is sparse. Future research should focus on validating existing models utilising TRIPOD guidelines to improve reporting quality. Future studies should also look to utilise decision curve analysis to analyse the net benefit of using the predictive model.[55 60]

For peer review only

For peer review only - http://bmjopen.bmj.com/site/about/guidelines.xhtml

Author Contributions:

TPK co-designed the protocol, undertook all stages of the review and co-wrote the paper. RPWK undertook all stages of the review and co-wrote the paper. MA undertook all stages of the review and co-wrote the paper. AB co-designed the protocol undertook all stages of the review and co-wrote the paper, VK undertook all stages of the review and co-wrote the paper. DC undertook all stages of the review and co-wrote the paper. CR undertook the searches for the review and co-wrote the paper. CHE undertook the searches for the review and co-wrote the paper. FRB co-designed the protocol and co-wrote the paper. FP contributed to the analysis of review data and co-wrote the paper. DC co-designed the protocol and co-wrote the paper. PL co-designed the protocol and co-wrote the paper. LV co-designed the protocol and co-wrote the paper. RE co-designed the protocol, undertook all stages of the review and co-wrote the paper.

Acknowledgements:

We would like to thank our colleagues from the Evidence Synthesis Group, and NIHR Innovation Observatory, of Newcastle University and Dr. Batoul Nasr from Dermatological Sciences, Translational Research Institute, Newcastle University for their support in screening.

Data availability statement:

All data relevant to the study are included in the article or uploaded as supplemental information.

Ethical approval:

This research was a review of published literature therefore ethics approval and participant consent were not required.

For peer review only

Figure 1: Identified Predictors

References

1. Matthews NH, Li WQ, Qureshi AA, et al. Epidemiology of melanoma. In: Ward WH, Farma JM, eds. Cutaneous melanoma: etiology and therapy. Brisbane (AU): Codon Publications 2017.
2. de Vries E, Sierra M, Pineros M, et al. The burden of cutaneous melanoma and status of preventive measures in Central and South America. *Cancer Epidemiol* 2016;44 Suppl 1:S100-s09. doi: 10.1016/j.canep.2016.02.005 [published Online First: 2016/04/02]
3. Svedman FC, Pillas D, Taylor A, et al. Stage-specific survival and recurrence in patients with cutaneous malignant melanoma in Europe - a systematic review of the literature. *Clin Epidemiol* 2016;8:109-22. doi: 10.2147/CLEP.S99021
4. Balch CM, Gershenwald JE, Soong S-J, et al. Final version of 2009 AJCC melanoma staging and classification. *J Clin Oncol* 2009;27(36):6199-206.
5. Thomson DR, Rughani MG, Kuo R, et al. Sentinel node biopsy status is strongly predictive of survival in cutaneous melanoma: Extended follow-up of Oxford patients from 1998 to 2014. *J Plast Reconstr Aesthet Surg* 2017;70(10):1397-403. doi: 10.1016/j.bjps.2017.05.025 [published Online First: 2017/06/20]
6. Gershenwald JE, Ross MI. Sentinel-lymph-node biopsy for cutaneous melanoma. *N Engl J Med* 2011;364(18):1738-45.
7. Barbour A, Millward M, Morton R, et al. Clinical practice guidelines for the diagnosis and management of melanoma Sydney: Cancer Council Australia: Clinical Guidelines Network; 2018 [cited 2019 11 October]. Available from: https://wiki.cancer.org.au/australia/Guidelines:Melanoma accessed 11 October 2019.
8. American Joint Committee on Cancer. AJCC Cancer Staging Manual. 8th ed. Chicago: American Joint Committee on Cancer/Springer 2017.
9. American Joint Committee on Cancer. AJCC Cancer Staging Manual. 7th ed. Chicago: American Joint Committee on Cancer 2010.
10. Moons KG, Kengne AP, Grobbee DE, et al. Risk prediction models: II. External validation, model updating, and impact assessment. *Heart* 2012;98(9):691-98.
11. Usher-Smith JA, Emery J, Kassianos AP, et al. Risk prediction models for melanoma: a systematic review. *Cancer Epidemiol Biomarkers Prev* 2014;23(8):1450-63.
12. Vuong K, McGeechan K, Armstrong BK, et al. Risk prediction models for incident primary cutaneous melanoma: a systematic review. *JAMA Dermatol* 2014;150(4):434-44.
13. Kaiser I, Pfahlberg AB, Uter W, et al. Risk prediction models for melanoma: a systematic review on the heterogeneity in model development and validation. *International Journal of Environmental Research and Public Health* 2020;17(21):7919.
14. Mahar AL, Compton C, Halabi S, et al. Critical assessment of clinical prognostic tools in melanoma. *Ann Surg Oncol* 2016;23(9):2753-61.
15. Cancer Research UK. Treatment for stage 1 and 2 melanoma 2022 [cited 2022 21 June]. Available from: chrome-extension://efaidnbmnnnibpcajpcglclefindmkaj/https://www.nhs.uk/ipgmedia/National/Cancer%20Research%20UK/assets/Treatingearly(Stage1)melanoma(CRUK2pages).pdf accessed 21 June 2022.
16. Moher D, Liberati A, Tetzlaff J, et al. Preferred reporting items for systematic reviews and meta-analyses: the PRISMA statement. *J Clin Epi* 2009;62(10):1006-12. doi: https://doi.org/10.1016/j.jclinepi.2009.06.005
17. Geersing G-J, Bouwmeester W, Zuithoff P, et al. Search filters for finding prognostic and diagnostic prediction studies in Medline to enhance systematic reviews. *PLoS One* 2012;7(2):e32844.

For peer review only - http://bmjopen.bmj.com/site/about/guidelines.xhtml

18. Hayden JA, Wilson MN, Riley RD, et al. Individual recovery expectations and prognosis of outcomes in non-specific low back pain: Prognostic factor review. *Cochrane Database Syst Rev* 2019(11)
19. Whitlock EP, Lopez SA, Chang S, et al. AHRQ Series Paper 3: Identifying, selecting, and refining topics for comparative effectiveness systematic reviews: AHRQ and the Effective Health-Care program. *J Clin Epi* 2010;63(5):491-501. doi: https://doi.org/10.1016/j.jclinepi.2009.03.008
20. Moons KG, de Groot JA, Bouwmeester W, et al. Critical appraisal and data extraction for systematic reviews of prediction modelling studies: the CHARMS checklist. *PLoS Med* 2014;11(10):e1001744.
21. Wolff RF, Moons KGM, Riley RD, et al. PROBAST: A tool to assess the risk of bias and applicability of prediction model studies (Prediction model Risk Of Bias ASsessment Tool). *Ann Intern Med* 2019;170(1):51-58. doi: 10.7326/m18-1376
22. de Jong Y, Ramspek CL, Zoccali C, et al. Appraising prediction research: a guide and meta-review on bias and applicability assessment using the Prediction model Risk Of Bias ASsessment Tool (PROBAST). *Nephrology* 2021;26(12):939-47.
23. Schünemann H, Brożek J, Guyatt G, et al. GRADE handbook for grading quality of evidence and strength of recommendations 2013 [cited 2020 18 December]. Available from: https://gdt.gradepro.org/app/handbook/handbook.html accessed 18 December 2020.
24. Iorio A, Spencer FA, Falavigna M, et al. Use of GRADE for assessment of evidence about prognosis: rating confidence in estimates of event rates in broad categories of patients. *BMJ* 2015;350:h870.
25. Baade PD, Royston P, Youl PH, et al. Prognostic survival model for people diagnosed with invasive cutaneous melanoma. *BMC Cancer* 2015;15:27.
26. Balch CM, Soong SJ, Gershenwald JE, et al. Prognostic factors analysis of 17,600 melanoma patients: Validation of the American Joint Committee on Cancer melanoma staging system. *J Clin Oncol* 2001;19(16):3622-34.
27. Cochran AJ, Elashoff D, Morton DL, et al. Individualized prognosis for melanoma patients. *Hum Pathol* 2000;31(3):327-31.
28. El Sharouni M-A, Ahmed T, Varey AH, et al. Development and validation of nomograms to predict local, regional, and distant recurrence in patients with thin (T1) melanomas. *J Clin Oncol* 2021;39(11):1243-52.
29. Fonseca IB, Lindote MVN, Monteiro MR, et al. Sentinel node status is the most important prognostic information for clinical stage IIB and IIC melanoma patients. *Ann Surg Oncol* 2020;27(11):4133-40.
30. Gimotty PA, Elder DE, Fraker DL, et al. Identification of high-risk patients among those diagnosed with thin cutaneous melanomas. *J Clin Oncol* 2007;25(9):1129-34.
31. Gimotty PA, Guerry D, Ming ME, et al. Thin primary cutaneous malignant melanoma: a prognostic tree for 10-year metastasis is more accurate than American Joint Committee on Cancer staging. *J Clin Oncol* 2004;22(18):3668-76.
32. Maurichi A, Miceli R, Camerini T, et al. Prediction of survival in patients with thin melanoma: results from a multi-institution study. *J Clin Oncol* 2014;32(23):2479-85.
33. Rosenbaum BE, Schafer C, Han SW, et al. Computer-assisted image analysis demonstrates tumor area and width as prognostic factors in stage IB melanoma. *Pigment Cell Melanoma Res* 2017;30(1):138.
34. Soong SJ, Ding S, Coit D, et al. Predicting survival outcome of localized melanoma: An electronic prediction tool based on the AJCC melanoma database. *Ann Surg Oncol* 2010;17(8):2006-14.
35. Tsai CA, Chen DT, Chen JJ, et al. An integrated tree-based classification approach to prognostic grouping with application to localized melanoma patients. *J Biopharm Stat* 2007;17(3):445-60.
36. Verver D, van Klaveren D, Franke V, et al. Development and validation of a nomogram to predict recurrence and melanoma-specific mortality in patients with negative sentinel lymph nodes. *Journal of British Surgery* 2019;106(3):217-25.

For peer review only - http://bmjopen.bmj.com/site/about/guidelines.xhtml

37. Vollmer RT, Seigler HF. Using a continuous transformation of the breslow thickness for prognosis in cutaneous melanoma. *Am J Clin Pathol* 2001;115(2):205-12.
38. Xiao Y, Peng S, Hu Y, et al. Development and validation of prognostic nomogram in patients with nonmetastatic malignant melanoma: a SEER population-based study. *Cancer Medicine* 2020;9(22):8562-70.
39. Debray TP, Koffijberg H, Nieboer D, et al. Meta-analysis and aggregation of multiple published prediction models. *Stat Med* 2014;33(14):2341-62.
40. Austin PC, Steyerberg EW. Graphical assessment of internal and external calibration of logistic regression models by using loess smoothers. *Stat Med* 2014;33(3):517-35.
41. Steyerberg EW. Clinical prediction models. New York: Springer 2009.
42. Vickers AJ, Van Calster B, Steyerberg EW. Net benefit approaches to the evaluation of prediction models, molecular markers, and diagnostic tests. *BMJ* 2016;352
43. Steyerberg EW, Harrell Jr FE, Borsboom GJ, et al. Internal validation of predictive models: efficiency of some procedures for logistic regression analysis. *J Clin Epi* 2001;54(8):774-81.
44. Debray TP, Vergouwe Y, Koffijberg H, et al. A new framework to enhance the interpretation of external validation studies of clinical prediction models. *J Clin Epi* 2015;68(3):279-89.
45. Wong T-T. Performance evaluation of classification algorithms by k-fold and leave-one-out cross validation. *Pattern Recognition* 2015;48(9):2839-46.
46. Johansson I, Tempel D, Dwarkasing J, et al. Validation of a clinicopathological and gene expression profile model to identify patients with cutaneous melanoma where sentinel lymph node biopsy is unnecessary. *Eur J Surg Oncol* 2022;48(2):320-25.
47. Buja A, Bardin A, Damiani G, et al. Prognosis for Cutaneous Melanoma by Clinical and Pathological Profile: A Population-Based Study. *Frontiers in Oncology* 2021;11:737399-99.
48. Steyerberg EW, Vickers AJ, Cook NR, et al. Assessing the performance of prediction models: a framework for some traditional and novel measures. *Epidemiology* 2010;21(1):128.
49. Vale L, Kunonga P, Coughlan D, et al. Optimal surveillance strategies for patients with stage 1 cutaneous melanoma post primary tumour excision: three systematic reviews and an economic model. *Health Technology Assessment* 2021
50. Kaiser I, Pfahlberg AB, Mathes S, et al. Inter-Rater Agreement in Assessing Risk of Bias in Melanoma Prediction Studies Using the Prediction Model Risk of Bias Assessment Tool (PROBAST): Results from a Controlled Experiment on the Effect of Specific Rater Training. *Journal of Clinical Medicine* 2023;12(5):1976.
51. Collins GS, Reitsma JB, Altman DG, et al. Transparent reporting of a multivariable prediction model for individual prognosis or diagnosis (TRIPOD): the TRIPOD statement. *BMC Med* 2015;13(1):1.
52. Reporting quality of studies developing and validating melanoma prediction models: an assessment based on the TRIPOD statement. Healthcare; 2022. MDPI.
53. Michaelson J. Melanoma Conditional Outcome Calculator 2011 [cited 2022 13 June]. Available from: http://lifemath.net/cancer/melanoma/condsurv/index.php accessed 13 June 2022.
54. Michaelson J. Melanoma Outcome Calculator 2011 [cited 2022 13 June]. Available from: http://lifemath.net/cancer/melanoma/outcome/index.php accessed 13 June 2022.
55. Vickers AJ, van Calster B, Steyerberg EW. A simple, step-by-step guide to interpreting decision curve analysis. *Diagn Progn Res* 2019;3(1):1-8.
56. Ding L, Gosh A, Lee DJ, et al. Prognostic biomarkers of cutaneous melanoma. *Photodermatology, photoimmunology & photomedicine* 2022;38(5):418-34.
57. Liu R, Lv Q-L, Yu J, et al. Correlating transcriptional networks with pathological complete response following neoadjuvant chemotherapy for breast cancer. *Breast Cancer Research and Treatment* 2015;151:607-18.
58. Adler NR, Wolfe R, Kelly JW, et al. Tumour mutation status and sites of metastasis in patients with cutaneous melanoma. *Br J Cancer* 2017;117(7):1026-35.

For peer review only - http://bmjopen.bmj.com/site/about/guidelines.xhtml

59. Calomarde-Rees L, García-Calatayud R, Caballero CR, et al. Risk factors for lymphatic and hematogenous dissemination in patients with stages I to II cutaneous melanoma. *JAMA Dermatol* 2019;155(6):679-87.
60. Sadatsafavi M, Adibi A, Puhan M, et al. Moving beyond AUC: decision curve analysis for quantifying net benefit of risk prediction models. *European Respiratory Journal* 2021;58(5)

For peer review only

For peer review only - http://bmjopen.bmj.com/site/about/guidelines.xhtml

Table 1: Inclusion criteria

Criteria	Include	Exclude
Population	Adults aged ≥ 18 years treated for American Joint Committee on Cancer (7th Edition) stage I and II cutaneous melanoma<br>➢ stage IA (T1a ≤ 1mm thick); or<br>➢ stage IB (T1b with ulceration or mitoses ≤ 1 mm thick, or T2a 1.01 to 2.00mm thick and no ulceration); or<br>➢ stage IIA (1.01-2.00mm thick with ulceration, or 2.01-4.00mm thick without ulceration); or<br>➢ stage IIB (2.01-4.00mm thick with ulceration, or > 4.0mm without ulceration)<br>➢ stage IIC (>4.0mm with ulceration)	Advanced melanoma<br>Metastatic melanoma<br>Stage III<br>Stage IV
Types of prognostic models	➢ Nomogram<br>➢ Scoring system<br>➢ Equation<br>➢ Classification or decision trees<br>*Minimum of 2 clinicopathologic factors	Models built with predictive biomarker factors included<br>Models built with gene expression profiling<br>Diagnostic studies<br>Studies assessing only one predictor
Outcome measures	Primary: Overall survival<br>Secondary: recurrence (any site), metastases, prognostic performance of risk models	
Timing	Post-resection of the primary cutaneous tumour<br>➢ Diagnostic excision<br>➢ Re-excision, also called wide local excision (WLE)	Studies that are looking at treatment (e.g. therapy) of melanoma
Setting	Any setting (primary, secondary, or tertiary care)	
Study design	Retrospective cohort studies<br>Prospective cohort studies<br>Randomised controlled trials	Non-empirical studies

For peer review only

Table 2: Characteristics of included studies

Citation	Study location	Study design	Data source (no. of centres)	AJCC Disease stage/Clinical staging	Study interval	Development method
Baade 2015[25]	Australia	Retrospective	Population based Queensland cancer registry	2002 AJCC<br>94% Stages I-II	1995 - 2008	Multivariate analysis
Balch 2001[26]	USA	Retrospective	Population based AJCC database (13)	Stages I-II	Not reported	Multivariate analysis
Cochran 2000[27]	USA	Retrospective	John Wayne Cancer Institute Melanoma clinical database, Division of Surgical Oncology, UCLA	1988 AJCC<br>Not reported	1980 - 1990	Multivariate analysis
El Sharouni 2021[28]	Netherlands<br>Australia	Retrospective	DEV: PALGA, the Dutch Pathology Registry<br>VALID: Melanoma institute Australia	2009 AJCC<br>Stages IA-IB<br>IA (T1a) = 58.6%<br>IB (T1b) = 29%<br>T1nos = 12.4%	2000 - 2014	Multivariate analysis
Fonseca 2020[29]	Brazil	Retrospective	A. C. Camargo Cancer Center database	2009 AJCC<br>Stages IIB-IIC	2000 - 2014	Multivariate analysis
Gimotty 2004[31]	USA	Retrospective	DEV: SEER population based registry<br>VALID: New SEER patients	2002 AJCC<br>Stages IA-IB<br>IA (T1a) = 86%<br>IB (T1b) = 14%	DEV: 1972 - 1991<br>VALID: 1991 - 1995	Recursive partitioning
Gimotty 2007[30]	USA	Retrospective	DEV: SEER Registry<br>VALID: Clinical based PLC registry	2002 AJCC<br>Stages IA (T1a) –IB (T1b)	DEV: 1998 – 2001<br>VALID: 1972 - 2001	Recursive partitioning
Maurichi 2014[32]	Europe	Retrospective	European clinical based centres (6)	2009 AJCC<br>Stages IA-IB<br>T1a = 50.3%<br>T1b = 49.7%	1996 - 2004	Multivariate analysis
Rosenbaum 2017[33]	USA	Prospective	NYU clinico-pathological biospecimen database	2009 AJCC<br>Stage IB	2002 - 2014	Multivariate analysis
Soong 2010[34]	USA	Prospective	DEV: 2008 AJCC population based Melanoma Database (9)<br>VALID: Sydney Melanoma Unit, Australia	2008 AJCC<br>Stages I-II	DEV: 26% diagnosed after 2002<br>VALID: Not reported	Multivariate analysis
Tsai 2007[35]	USA	Prospective	AJCC Melanoma population-based database (13)	2002 AJCC<br>Stages IA-IIC	Not reported	Recursive partitioning

For peer review only

Citation	Study location	Study design	Data source (no. of centres)	AJCC Disease stage/Clinical staging	Study interval	Development method
Verver 2019[36]	Europe	Retrospective	EORTC	2009 AJCC Stages IA-IIC	1997 - 2013	Multivariate analysis
Vollmer 2001[37]	USA	Retrospective	University Melanoma Clinic database	1988 AJCC Not reported	1980 - 1990	Multivariate analysis
Xiao 2020[38]	USA	Retrospective	SEER Registry	2009 AJCC Stages IA-IIC	2010 - 2015	Multivariate analysis

Abbreviations: AJCC = American Joint Committee on Cancer; DEV = Development set; NYU = New York University; EORTC = European Organization for Research and Treatment of Cancer Centres SEER = Surveillance, Epidemiology, and End Results Program; UCLA = University of California, Los Angeles; VALID = Validation Set; RND = Regional Node Dissection

For peer review only

Table 3: Model development

Citation	Sample size	Follow up period	End point	Number of events	Candidate selection method	Final predictor selection	Number of final predictors	Validation method
Baade 2015[25]	28,654	Median = 7.2 years (86.4 months)	MSS	1,700	Pre-specification from existing data	Backward elimination	7	Internal-external cross validation
Balch 2001[26]	13,581	5 - 20 years	MSS	NR	Unclear	Unclear	8	External - geographic
Cochran 2000a[27]	1,042	Median = 42.5 months	OS	NR	Pre-specification + univariate analysis	Backward elimination	5	Internal sample validation
Cochran 2000b[27]	1042	Median = 42.5 months	Recurrence	NR	Pre-specification + univariate analysis	Backward elimination	4	Internal sample validation
El Sharouni 2021a[28]	DEV: 25,930 VALID: 2,968	DEV: Median = 6.7yrs VALID: Median = 12yrs	LRFS	232	Pre-specification from existing data	Backward elimination	6	Internal-external cross validation
El Sharouni 2021b[28]	DEV: 25,930 VALID: 2,968	DEV: Median = 6.7yrs VALID: Median = 12yrs	RRFS	564	Pre-specification from existing data	Backward elimination	7	Internal-external cross validation
El Sharouni 2021c[28]	DEV: 25,930 VALID: 2,968	DEV: Median = 6.7yrs VALID: Median = 12yrs	DRFS	278	Pre-specification from existing data	Backward elimination	6	Internal-external cross validation
Fonseca 2020a[29]	259	Median = 80.13 months	DRFS	117	Univariate analysis	Backward elimination	5	Bootstrap
Fonseca 2020b[29]	259	Median = 80.13 months	MSS	117	Univariate analysis	Backward elimination	5	Bootstrap
Gimott y 2004[31]	DEV: 884 VALID: 114	At least 10 years	Metastasis	127	Multivariate analysis	Backward elimination	4	External – new cohort

For peer review only

Citation	Sample size	Follow up period	End point	Number of events	Candidate selection method	Final predictor selection	Number of final predictors	Validation method
Gimotty 2007a[30]	DEV: 26,114	Median = 4.6 years	OS	3,593	Pre-specification from existing data	Full model approach	4	External - geographic
Gimotty 2007b[30]	VALID: 2,389	Median = 8.1 years	OS	1076	Pre-specification from existing data	Full model approach	5	External - geographic
Maurichi 2014[32]	2,243	124 months	12-year OS	240	Multivariate analysis	Full model approach	6	Congruence examination
Rosenbaum 2017[33]	DEV: 506 VALID: 149	Median = 4.4 years	RFS	NR	Univariate analysis	Backward elimination	3	Internal K-fold validation
Soong 2010[34]	DEV: 14,760 VALID: 10,974	5 and 10 years	OS	NR	Multivariate analysis	Full model approach	6	External - geographic
Tsai 2007[35]	13,268	Not reported	OS	NR	Pre-specification from existing data	Full model approach	6	Internal-external cross validation
Verver 2019[36]	3180	Median = 61 months	Composite Recurrence and/or MSM	Recurrence = 496 Dead = 277	Pre-specification + univariate analysis	Backward elimination	3	Internal-external cross validation
Vollmer 2001[37]	1,910	Median = 7.6 years	Mortality	NR	Pre-specification	Full model approach	5	Internal K-fold validation
Xiao 2020[38]	DEV: 46,336 VALID: 19,856	3 and 5 years	OS	NR	Pre-specification + univariate analysis	Full model approach	10	Internal sample validation

Abbreviations: DEV = Development set; VALID = Validation set; MSS = Melanoma Specific Survival; OS = Overall Survival; DRFS = Distant Recurrence-Free survival; RFS = Recurrence Free Survival; RRFS = Regional Recurrence Free Survival; MSM = Melanoma Specific Mortality; NR = Not Reported; EPV = Events Per Variable

For peer review only - http://bmjopen.bmj.com/site/about/guidelines.xhtml

Supplementary Materials

Supplemental Table 1: Medline Search Strategy

Supplemental Table 2: Updated Medline Search Strategy

Supplemental Table 3: List of databases

Supplemental Table 4: Definitions for the performance measures

Supplemental Figure 1: PRISMA Flow Diagram

Supplemental Table 5: Model outcome definitions

Supplemental Table 6: Model evaluation

Supplemental Table 7: Risk of bias assessment

Supplemental Table 8: Model Applicability Assessment

Supplemental File 1: Risk of bias assessment

PRISMA Checklist

For peer review only

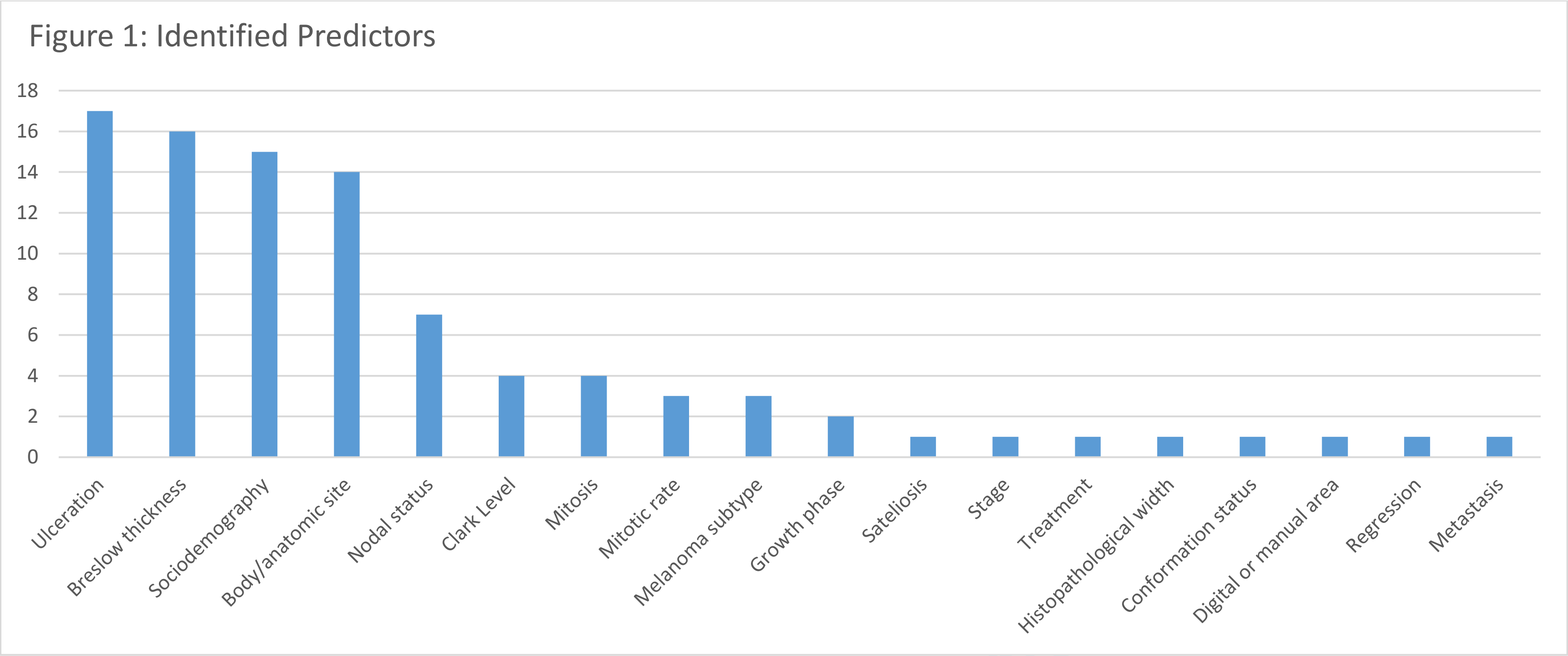
Figure 1: Identified Predictors
18
16
14
12
10
8
6
4
2
0
Ulceration
Breslow thickness
Sociodemography
Body/anatomic site
Nodal status
Clark Level
Mitosis
Mitotic rate
Melanoma subtype
Growth phase
Sateliosis
Stage
Treatment
Histopathological width
Conformation status
Digital or manual area
Regression
Metastasis

Supplementary Materials

Supplemental Table 1: Medline Search Strategy

Originally searched September 2018, update searches in 16th July 2019, and 30th September 2021

Database(s): Ovid MEDLINE(R) and In-Process & Other Non-Indexed Citations 1946 to July 15, 2019

#	Searches	Results
1	(Validat* or Predict* or Rule* or (Predict* and (Outcome* or Risk* or Model*)) or ((History or Variable* or Criteria or Scor* or Characteristic* or Finding* or Factor*) and (Predict* or Model* or Decision* or Identif* or Prognos*))).mp. or (Decision*.mp. and ((Model* or Clinical*).mp. or Logistic Models/)) or (Prognostic and (History or Variable* or Criteria or Scor* or Characteristic* or Finding* or Factor* or Model*)).ti,ab. [mp=title, abstract, original title, name of substance word, subject heading word, floating sub-heading word, keyword heading word, organism supplementary concept word, protocol supplementary concept word, rare disease supplementary concept word, unique identifier, synonyms]	4600871
2	Stratification.mp. or ROC Curve/ or Discrimination.mp. or Discriminate.mp. or c-statistic.mp. or c statistic.mp. or Area under the curve.mp. or AUC.mp. or Calibration.mp. or Indices.mp. or Algorithm.mp. or Multivariable.mp. or Prognosis.mp. [mp=title, abstract, original title, name of substance word, subject heading word, floating sub-heading word, keyword heading word, organism supplementary concept word, protocol supplementary concept word, rare disease supplementary concept word, unique identifier, synonyms]	1450061
3	1 or 2	5285509
4	*melanoma/	65466
5	*skin neoplasms/	99710
6	melanoma.kw.	7937
7	(malignant adj3 melanoma*).ti,ab.	27244
8	(tumo* adj5 (mole or melanoma)).ti,ab.	12160
9	4 or 5 or 6 or 7 or 8	149902
10	(((prognos* or melanoma) adj5 surviv*) or factor*).ti,ab.	3091009
11	(metas* or advance* or recur* or relaps* or invasive or second* or disseminat*).ti,ab.	3355846
12	(distant metastases or local recurren*).ti,ab.	45561
13	10 or 11 or 12	5901577
14	exp comment/ or exp letter/ or exp editorial/	1731981

For peer review only - http://bmjopen.bmj.com/site/about/guidelines.xhtml

#	Searches	Results
15	exp animals/ not exp humans/	4595710
16	(animal or mouse).mp. or mice.ti,ab. [mp=title, abstract, original title, name of substance word, subject heading word, floating sub-heading word, keyword heading word, organism supplementary concept word, protocol supplementary concept word, rare disease supplementary concept word, unique identifier, synonyms]	2072446
17	exp review/	2504343
18	exp case reports/ or case report*.ti,ab.	2103512
19	or/14-18	10922034
20	(3 and 9 and 13) not 19	13880
21	(201808* or 201809* or 20181* or 2019*).ed. or 2019*.dp.	1423830
22	20 and 21	1023

For peer review only

Supplemental Table 2: Updated Medline Search Strategy

Updated search for the period June 2019 to current. Conducted on 30th September 2021

Database(s): Ovid MEDLINE(R) <1946 to September Week 4 2021>

#	Searches	Results
1	Melanoma/	89251
2	melano*.ti,ab,kw.	176833
3	(malignant adj3 melanoma*).ti,ab,kw.	26917
4	skin neoplasms/	127346
5	(tumo* adj5 (mole or melanoma)).ti,ab.	12439
6	1 or 2 or 3 or 4 or 5	271222
7	(ocular or uveal or iris or cornea or eye or choroidal or ciliary or intraocular).ti.	161269
8	6 not 7	262830
9	(metas* or advance* or recur* or relaps* or invasive or second*).ti,ab,kw.	3286319
10	(distant metastases or local recurren*).ti,ab.	45864
11	9 or 10	3286319
12	Cohort Studies/	294363
13	incidence.tw.	705176
14	Mortality/	47593
15	Follow-Up Studies/	671388
16	prognos*.tw.	568943
17	predict*.tw.	1442443
18	course.tw.	523515
19	Survival Analysis/	141766
20	12 or 13 or 14 or 15 or 16 or 17 or 18 or 19	3641777
21	8 and 11 and 20	22371
22	exp case reports/ or case report*.ti,ab,kw.	2112036
23	exp comment/ or exp letter/ or exp editorial/	1835116
24	exp review/	2617551
25	exp animals/ not humans.sh.	4889865
26	22 or 23 or 24 or 25	10795089
27	21 not 26	14321

For peer review only - http://bmjopen.bmj.com/site/about/guidelines.xhtml

#	Searches	Results
28	(201906* or 201907* or 201908* or 201909* or 201910* or 201911* or 201912* or "2020" or 2021*).ed. or (2019* or 2020* or 2021*).dp.	2635276
29	27 and 28	2267

For peer review only

For peer review only - http://bmjopen.bmj.com/site/about/guidelines.xhtml

Supplemental Table 3: List of databases

- MEDLINE (OVID)
- EMBASE (OVID)
- CENTRAL (Cochrane Library via Wiley)
- Health Technology Assessment database (Cochrane Library via Wiley)
- CINAHL[a] (EBSCO)
- Science Citation Index (Web of Science)
- Conference Proceedings Citation Index – Science (Web of Science)
- Cochrane Database of Systematic Reviews (Cochrane Library via Wiley – to check included studies of relevant reviews).
- Grey literature was sought using similar keywords to search various resources including but not limited to the following:
- OpenGrey (http://www.opengrey.eu/, includes SIGLE, EAGLE, GreyNet)
- Cancer Research UK (http://www.cancerresearchuk.org/about-cancer/find-a-clinical-trial)
- Melanoma UK (http://www.melanomauk.org.uk)
- National Guideline ClearingHouse (https://www.guideline.gov/)
- Ongoing trials identified using the WHO ICTRP platform of trials registries. (www.who.int/trialsearch).

For peer review only

For peer review only - http://bmjopen.bmj.com/site/about/guidelines.xhtml

Supplemental Table 4: Definitions for the performance measures

Performance measure	Definition	Statistic/Measure	Presentation	Performance criteria
Discrimination[35,37]	How well a prediction model can discriminate those with the outcome from those without the outcome	C-statistic	ROC curve	Estimates are interpreted as the area under the receiver operating characteristics curve (AUC-ROC), with values between 0.5 (no discriminative ability) to 1 (perfect discriminative ability)
		Discrimination slope	Box plot	
		Harrell C statistic/ concordance statistic	ROC curve	
		Gonen and Heller K-statistic		
Calibration[35,37]	A model's accuracy of predicted risk probabilities and indicates the extent to which expected outcomes (predicted from the model) and observed outcomes agree	Calibration slope	Calibration or validation graph	Calibration can be reported as scores range from 0 (no agreement) to 1 (perfect agreement), or as plots showing the relation between the estimated risk (on the x-axis) and the observed proportion of events (y-axis). A curve close to the diagonal indicates that predicted risks correspond well to observed proportions.
		Calibration-in-the-large	Calibration or validation graph	
		Calibration test	Calibration graph	
		Hosmer-Lemeshow test	Calibration or validation graph	
		Harrell's E Statistic	Calibration graph	
Overall performance[35,37]	The distance between the predicted and observed outcomes	Brier score	Validation graph	Considered as very weak (R2 of 0 to 4%), weak (4 to 16%), moderate (16 to 36%), strong (36 to 64%) or very strong (64% to 100%).
		R-squared statistic	Validation graph	
Clinical utility[44,50]	Defined as the minimum probability of disease at which further intervention would be warranted	Decision curve analysis	Decision curve	Net benefit = sensitivity × prevalence – (1 – specificity) × (1 – prevalence) × w where w is the odds at the threshold probability.
		Net benefit	Cross table	

For peer review only - http://bmjopen.bmj.com/site/about/guidelines.xhtml

Supplemental Figure 1: PRISMA Flow Diagram

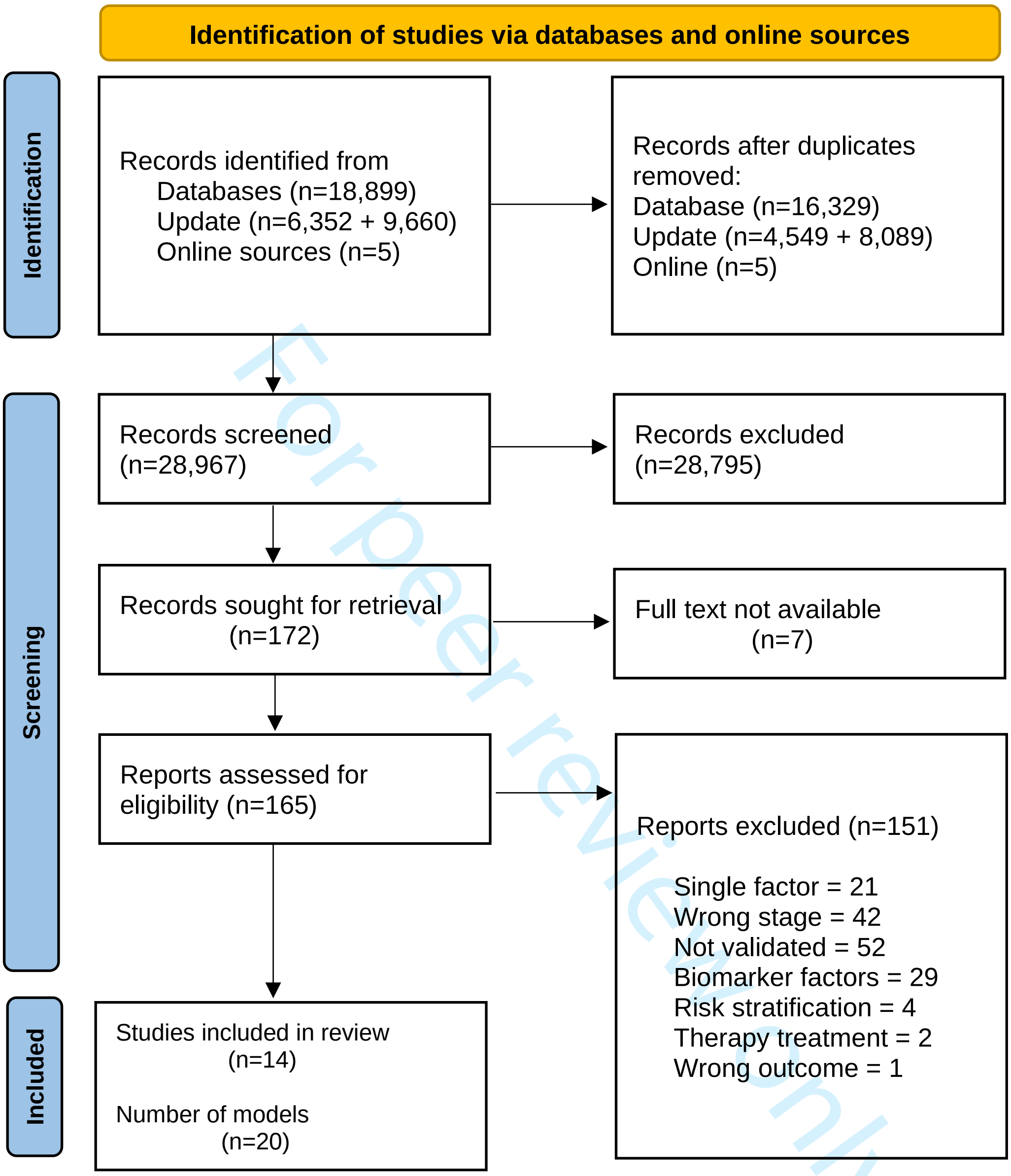

For peer review only

Supplemental Table 5: Model Outcome definitions

Citation	Outcome	Definition provided by authors
Baade 2015[25]	MSS	Melanoma-specific survival was estimated from the mortality of people diagnosed with melanoma between 1995 and 2008 (inclusive), with follow-up for all cases to December 31, 2010.
Balch 2001[26]	MSS	Survival times were calculated from onset of primary melanoma diagnosis and considered censored for patients who were alive at the last follow-up or who died without evidence of melanoma.
Cochran 2000a[27]	OS	Not reported
Cochran 2000b[27]	Recurrence	Not reported
El Sharouni 2021a[28]	LRFS	Recurrence free-survival was calculated from the date of initial melanoma diagnosis to the date of diagnosis of recurrence.
El Sharouni 2021b[28]	RRFS	Recurrence free-survival was calculated from the date of initial melanoma diagnosis to the date of diagnosis of recurrence.
El Sharouni 2021c[28]	DRFS	Recurrence free-survival was calculated from the date of initial melanoma diagnosis to the date of diagnosis of recurrence.
Fonseca 2020a[29]	DFRS	Considered from the date when SNB was performed to the date of the event of interest.
Fonseca 2020b[29]	MSS	Considered from the date when SNB was performed to the date of the event of interest.
Gimotty et al. 2004[31]	Metastasis	Not reported
Gimotty 2007a[30]	OS	Survival time for SEER patients was defined as the time between diagnosis and melanoma-related death
Gimotty 2007b[30]	OS	Survival time for PLG patients was defined as the time between definitive surgical treatment and melanoma-related death.
Maurichi 2014[32]	OS	Overall survival (OS) was calculated from date of surgery for primary melanoma to date of death as a result of all causes or censored at date of last follow-up in living patients.
Rosenbaum 2017[33]	RFS	Recurrence-free survival was defined as the time from the date of pathological diagnosis of stage IB melanoma to the first recorded date of regional or distant metastases
Soong 2010[34]	OS	Survival times were calculated from onset of primary melanoma diagnosis and considered censored for patients who were alive at the last follow-up or who died without evidence of melanoma
Tsai 2007[35]	OS	Not reported

For peer review only - http://bmjopen.bmj.com/site/about/guidelines.xhtml

Citation	Outcome	Definition provided by authors
Verver 2019a[36]	Recurrence	Calculated from date of SLNB to date of first recurrence
Verver 2019b[36]	MSM	Calculated from date of SLNB to date of first death
Vollmer 2001[37]	MSS	Not reported
Xiao 2020[38]	OS	Not reported

Abbreviations: ROB = Risk of Bias; MSS = Melanoma Specific Survival; OS = Overall Survival; DRFS = Distant Recurrence-Free survival; RFS = Recurrence Free Survival; RRFS = Regional Recurrence Free Survival; MSM = Melanoma Specific Mortality; SEER = Surveillance, Epidemiology, and End Results Program; PLG = University of Pennsylvania's Pigmented Lesion Group; SLNB = Sentinel Lymph Node Biopsy; SNB = Sentinel Node Biopsy

For peer review only

For peer review only - http://bmjopen.bmj.com/site/about/guidelines.xhtml

Supplemental Table 6: Model evaluation

Citation	Predictors in final model	Type of model	Model performance			Validation method
			Discrimination	Calibration	Overall Performance	
Baade 2015[25]	Age, sex, smooth-rank transform thickness, body site, ulceration, positive lymph nodes, metastasis	Melanoma Severity Index	D-statistic = 1.50 (1.44 to 1.56) Harrell-C statistic = 0.88 (0.88 to 0.89)	NA	RD2 statistic = 0.47 (0.45 to 0.49)	Internal-external cross validation
Balch 2001[26]	Age, sex, ulceration, tumour thickness, anatomic level, nodal status, number of metastatic nodes, site	AJCC Validation	NA	NA	NA	External (geographical)
Cochran 2000a[27]	Age, sex, ulceration, site, Breslow thickness	OS Risk Score	NA	NA	NA	Internal (random split)
Cochran 2000b[27]	Ulceration, site, Breslow thickness, melanoma subtype	Recurrence Risk Score	NA	NA	NA	Internal (random split)
El Sharouni 2021a[28]	Age, ulceration, site, Breslow thickness, melanoma subtype, mitosis	LRFS Nomogram	C-statistic DEV: 0.79 (0.76 to 0.82) VALID: 0.80 (0.69 to 0.90)	Calibration plot	NA	Internal (bootstrap) + external (geographical)
El Sharouni 2021b[28]	Sex, ulceration, site, Breslow depth, melanoma subtype, SLN status, mitosis	RRFS Nomogram	C-statistic DEV: 0.77 (0.75 to 0.79) VALID: 0.76 (0.70 to 0.82)	Calibration plot	NA	Internal (bootstrap) + external (geographical)
El Sharouni 2021c[28]	Sex, ulceration, site, Breslow depth, SLN status, mitosis	DRFS Nomogram	C-statistic DEV: 0.80 (0.77 to 0.83) VALID: 0.74 (0.69 to 0.80)	Calibration plot	NA	Internal (bootstrap) + external (geographical)

For peer review only - http://bmjopen.bmj.com/site/about/guidelines.xhtml

Citation	Predictors in final model	Type of model	Model performance			Validation method
			Discrimination	Calibration	Overall Performance	
Fonseca 2020a[29]	Age, ulceration, Breslow thickness, mitotic rate, SLN status	DRFS Nomogram	Harrell C-index = 0.734	Calibration plot	NA	Internal (bootstrap)
Fonseca 2020b[29]	Ulceration, Breslow thickness, mitotic rate, SLN status, satellitosis	MSS Nomogram	Harrell C-index = 0.718	Calibration plot	NA	Internal (bootstrap)
Gimotty 2004[31]	Sex, growth phase (vertical or radial), vertical growth phase (VGP), VGP tumour-infiltrating lymphocyte	Metastasis Prognostic classification tree	Risk groups: AUC = 0.85<br>AJCC Stage: AUC = 0.59	NA	NA	External (new cohort)
Gimotty 2007a[30]	Sex, body site, ulceration, tumour thickness, invasion level	OS Prognostic classification tree	SEER: AUC = 0.76	NA	NA	External (geographical)
Gimotty 2007b[30]	Age, sex, clark level, vertical growth mitogenicity	OS Prognostic classification tree	PLG: AUC = 0.83	NA	NA	External (geographical)
Maurichi 2014[32]	Age, ulceration, mitotic rate, lymphovascular invasion, regression, sentinel node status	OS Nomogram	Harrell C statistic = 0.88	Calibration plot	NA	Internal
Rosenbaum 2017[33]	Histopathological width, conformation status (contiguous or noncontiguous), digital or manual area	RFS Nomogram	Youden Index of the AU-ROC curve: AUC = 0.733 (0.647 to 0.818)	NA	NA	Internal (ten-fold cross-validation)
Soong 2010[34]	Age, ulceration, tumour thickness, site, invasion level, lesion site	MSS nomogram<br><br>Web-based tool/ melanomaprognosis.org	NA	Correlation coefficients:<br>5 years = 0.90<br>10 years = 0.93	NA	External (geographical)

For peer review only - http://bmjopen.bmj.com/site/about/guidelines.xhtml

Citation	Predictors in final model	Type of model	Model performance			Validation method
			**Discrimination**	**Calibration**	**Overall Performance**	
Tsai 2007[35]	Age, sex, ulceration, tumour thickness, site, invasion level	Prognostic classification tree	NA	NA	Brier score<br>At 1 year: 0.02<br>At 15 years: 0.20	Internal (cross-validation)
Verver 2019a[36]	Ulceration, anatomical location and Breslow thickness	Recurrence Nomogram	C-index = 0.74	Calibration slope 1.10 [0.96 to 1.24]	NA	Internal-external cross validation
Verver 2019b[36]	Ulceration, anatomical location and Breslow thickness	MSM Nomogram	C-index = 0.76		NA	Internal-external cross validation
Vollmer 2001[37]	Age, sex, ulceration, tumour thickness, site	Scoring system	NA	NA	NA	Internal (two-fold cross-validation)
Xiao 2020[38]	Age, sex, race, marital status, anatomic site, stage, depth, ulceration, mitoses, treatment	OS Nomogram	C-index<br>DEV: 0.817 (0.811 to 0.823)<br>VALID: 0.817 (0.809 to 0.825)	Calibration plot	NA	Internal (split sample)

Abbreviations: MSS = Melanoma Specific Survival; OS = Overall Survival; DRFS = Distant Recurrence-Free survival; RFS = Recurrence Free Survival;

RRFS = Regional Recurrence Free Survival; AU-ROC = Area under the Receiver Operating Characteristic

For peer review only

Supplemental Table 7: Risk of bias assessment

Study	Endpoint	ROB				Overall R.O.B
		Participants	Predictors	Outcome	Analysis	
Baade 2015[25]	MSS	+	?	+	?	?
Balch 2001[26]	MSS	+	+	+	?	?
Cochran 2000a[27]	OS	-	?	+	-	-
Cochran 2000b[27]	Recurrence	-	?	+	-	-
El Sharouni 2021a[28]	LRFS	+	?	+	+	?
El Sharouni 2021b[28]	RRFS	+	?	+	+	?
El Sharouni 2021c[28]	DRFS	+	?	+	+	?
Fonseca 2020a[29]	DFRS	+	?	+	-	-
Fonseca 2020b[29]	MSS	+	?	+	-	-
Gimotty et al. 2004[31]	Metastasis	+	?	+	+	?
Gimotty 2007a[30]	OS	+	+	+	?	?
Gimotty 2007b[30]	OS	+	+	+	?	?
Maurichi 2014[32]	OS	+	+	+	+	+
Rosenbaum 2017[33]	RFS	+	?	+	-	-
Soong 2010[34]	OS	+	+	+	+	+
Tsai 2007[35]	OS	+	+	+	+	+
Verver 2019a[36]	Recurrence	+	?	+	-	-
Verver 2019b[36]	MSM	+	?	+	-	-
Vollmer 2001[37]	MSM	+	+	+	?	?
Xiao 2020[38]	OS	+	?	+	-	-
+ Indicates low ROB/ low concern regarding applicability						
- Indicates high ROB/ high concern regarding applicability						
? Indicates unclear ROB/ unclear concern regarding applicability						

Abbreviations: ROB = Risk of Bias; MSS = Melanoma Specific Survival; OS = Overall Survival; DRFS = Distant Recurrence-Free survival; RFS = Recurrence Free Survival; RRFS = Regional Recurrence Free Survival; MSM = Melanoma Specific Mortality

For peer review only - http://bmjopen.bmj.com/site/about/guidelines.xhtml

Supplemental Table 8: Model Applicability Assessment

Study	Endpoint	Applicability			Overall Applicability
		Participants	Predictors	Outcome	
Baade 2015[25]	MSS	+	+	+	+
Balch 2001[26]	MSS	+	+	+	+
Cochran 2000a[27]	OS	-	+	+	-
Cochran 2000b[27]	Recurrence	-	+	+	-
El Sharouni 2021a[28]	LRFS	+	+	+	+
El Sharouni 2021b[28]	RRFS	+	+	+	+
El Sharouni 2021c[28]	DRFS	+	+	+	+
Fonseca 2020a[29]	DFRS	+	+	+	+
Fonseca 2020b[29]	MSS	+	+	+	+
Gimotty et al. 2004[31]	Metastasis	+	+	+	+
Gimotty 2007a[30]	OS	+	+	+	+
Gimotty 2007b[30]	OS	+	+	+	+
Maurichi 2014[32]	OS	+	+	+	+
Rosenbaum 2017[33]	RFS	+	+	+	+
Soong 2010[34]	OS	+	+	+	+
Tsai 2007[35]	OS	+	+	+	+
Verver 2019a[36]	Recurrence	+	+	+	+
Verver 2019b[36]	MSM	+	+	+	+
Vollmer 2001[37]	MSM	+	+	+	+
Xiao 2020[38]	OS	+	+	+	+
+ Indicates low ROB/ low concern regarding applicability					
- Indicates high ROB/ high concern regarding applicability					
? Indicates unclear ROB/ unclear concern regarding applicability					

Abbreviations: ROB = Risk of Bias; MSS = Melanoma Specific Survival; OS = Overall Survival; DRFS = Distant Recurrence-Free survival; RFS = Recurrence Free Survival; RRFS = Regional Recurrence Free Survival; MSM = Melanoma Specific Mortality

For peer review only - http://bmjopen.bmj.com/site/about/guidelines.xhtml

Supplemental File 1: Risk of bias assessment

Assessments were conducted using the PROBAST tool. Overall, eight models were judged to be at high risk of bias, three models were rated as low, and nine models were rated as having an unclear risk. Bias was introduced by various methods.

Selection of participants

Only two models (Cochran OS model,[27] and Cochran recurrence model[27]) were judged to be at high risk of bias. Very little detail was given on the participants in the study and there was no summary of patient characteristics to judge the severity of their melanoma. The rest of the studies were all classified as at low risk of bias.

Risk of bias introduced by predictors

Thirteen models: El Sharouni LRFS model;[28] El Sharouni RRFS model;[28] El Sharouni DRFS model;[28] Cochran OS model;[27] Cochran recurrence model;[27] Fonseca DRFS model;[29] Fonseca MSS model;[29] Verver recurrence model;[36] and Verver MSM model,[36] were rated to have an unclear risk of bias for selection of risk factors included in the final models. The models did not report information on blinding, and the predictors for these models were decided through backward variable selection procedure. With this method, variables are chosen based on significance level, meaning that variables regarded as insignificant, following univariate analysis, are removed from the model. However, the remaining seven models, were rated to have a low risk of bias for this domain. Although the model studies did not report information on blinding of predictor assessment to outcome data, their measurements can be inferred to be blinded to the outcome as predictors appeared to be measured and reported before the outcome occurred.

Risk of bias introduced by outcomes

All models had low concern regarding the timing of assessment. It was felt that the length of follow-up times for the outcomes assessed would not bias the prediction models.

Risk of bias introduced by the analysis

Eight models reported in five studies: Cochran OS model;[27] Cochran recurrence model;[27] Fonseca DRFS model;[29] Fonseca MSS model;[29] Verver recurrence model;[36] Verver MSM model;[36] Rosenbaum RFS model;[33] and Xiao OS model,[38] were judged to be of high risk of bias. This was mainly due to the

For peer review only - http://bmjopen.bmj.com/site/about/guidelines.xhtml

inclusion of variables identified as significant following a univariate analysis.[23,29,34] This method can lead to incorrect predictor selection because predictors are chosen on the basis of their statistical significance as a single predictor rather than in context with other predictors.[36,37] Dichotomization of continuous predictors was a concern for some of the models.[25,34] This can potentially reduce a model's predictive ability, by assuming a different risk for different groups.[36,37] Relevant information on whether model overfitting and optimism in model performance was accounted for was not reported in one model.[32]

Concerns regarding applicability

Overall, only the Cochran OS model,[27] and Cochran recurrence model,[27] were rated as having a high-risk regarding applicability, and the remaining nineteen models were rated as having a low risk regarding applicability.

Concern regarding selection of participants

The Cochran OS model,[27] and Cochran recurrence model,[27] were judged to have a high-risk of bias regarding selection of participants. Patients recruited for model development were a subset of the John Wayne Cancer Institute Melanoma database, however not much information was given regarding the characteristics of the patients or the severity of their melanoma. The rest of the models were rated as having a low concern because participant data were taken from routine care or cancer registries, the participants included in the studies matched the participants in the review question. Therefore, the characteristics of patients in the study were likely to match patients with similar characteristics presenting in either a different setting or country.

Concern regarding assessment and timing of predictors

All models had low concern regarding the definition, assessment, and timing of the predictors. All predictors were measured using methods potentially applicable to the daily practice that is addressed by the review.

Concern regarding the applicability of the outcome determined

All models were judged to have a low concern regarding the applicability of the outcomes. Our systematic review explicitly aimed to include all validations of the model regardless of outcome definition and measurement method.

For peer review only - http://bmjopen.bmj.com/site/about/guidelines.xhtml

For peer review only

PRISMA 2020 Checklist

Section and Topic	Item #	Checklist item	Location where item is reported
**TITLE**			
Title	1	Identify the report as a systematic review.	1
**ABSTRACT**			
Abstract	2	See the PRISMA 2020 for Abstracts checklist.	3-4
**INTRODUCTION**			
Rationale	3	Describe the rationale for the review in the context of existing knowledge.	5
Objectives	4	Provide an explicit statement of the objective(s) or question(s) the review addresses.	5-6
**METHODS**			
Eligibility criteria	5	Specify the inclusion and exclusion criteria for the review and how studies were grouped for the syntheses.	6
Information sources	6	Specify all databases, registers, websites, organisations, reference lists and other sources searched or consulted to identify studies. Specify the date when each source was last searched or consulted.	6 and Supplementary Table 3
Search strategy	7	Present the full search strategies for all databases, registers and websites, including any filters and limits used.	Supplementary Table 1 and Table 2
Selection process	8	Specify the methods used to decide whether a study met the inclusion criteria of the review, including how many reviewers screened each record and each report retrieved, whether they worked independently, and if applicable, details of automation tools used in the process.	6
Data collection process	9	Specify the methods used to collect data from reports, including how many reviewers collected data from each report, whether they worked independently, any processes for obtaining or confirming data from study investigators, and if applicable, details of automation tools used in the process.	7
Data items	10a	List and define all outcomes for which data were sought. Specify whether all results that were compatible with each outcome domain in each study were sought (e.g. for all measures, time points, analyses), and if not, the methods used to decide which results to collect.	6
	10b	List and define all other variables for which data were sought (e.g. participant and intervention characteristics, funding sources). Describe any assumptions made about any missing or unclear information.	6
Study risk of bias assessment	11	Specify the methods used to assess risk of bias in the included studies, including details of the tool(s) used, how many reviewers assessed each study and whether they worked independently, and if applicable, details of automation tools used in the process.	7
Effect measures	12	Specify for each outcome the effect measure(s) (e.g. risk ratio, mean difference) used in the synthesis or presentation of results.	7
Synthesis methods	13a	Describe the processes used to decide which studies were eligible for each synthesis (e.g. tabulating the study intervention characteristics and comparing against the planned groups for each synthesis (item #5)).	6-7
	13b	Describe any methods required to prepare the data for presentation or synthesis, such as handling of missing summary statistics, or data conversions.	7
	13c	Describe any methods used to tabulate or visually display results of individual studies and syntheses.	7
	13d	Describe any methods used to synthesize results and provide a rationale for the choice(s). If meta-analysis was performed, describe the model(s), method(s) to identify the presence and extent of statistical heterogeneity, and software package(s) used.	7
	13e	Describe any methods used to explore possible causes of heterogeneity among study results (e.g. subgroup analysis, meta-regression).	7
	13f	Describe any sensitivity analyses conducted to assess robustness of the synthesized results.	7
Reporting bias	14	Describe any methods used to assess risk of bias due to missing results in a synthesis (arising from reporting biases).	7

For peer review only

For peer review only - http://bmjopen.bmj.com/site/about/guidelines.xhtml

PRISMA 2020 Checklist

Section and Topic	Item #	Checklist item	Location where item is reported
assessment			
Certainty assessment	15	Describe any methods used to assess certainty (or confidence) in the body of evidence for an outcome.	7
**RESULTS**			
Study selection	16a	Describe the results of the search and selection process, from the number of records identified in the search to the number of studies included in the review, ideally using a flow diagram.	8
	16b	Cite studies that might appear to meet the inclusion criteria, but which were excluded, and explain why they were excluded.	Supplementary Fig.1
Study characteristics	17	Cite each included study and present its characteristics.	8
Risk of bias in studies	18	Present assessments of risk of bias for each included study.	11-12 and Supplementary File 1 (page 15-16)
Results of individual studies	19	For all outcomes, present, for each study: (a) summary statistics for each group (where appropriate) and (b) an effect estimate and its precision (e.g. confidence/credible interval), ideally using structured tables or plots.	9-10
Results of syntheses	20a	For each synthesis, briefly summarise the characteristics and risk of bias among contributing studies.	NA
	20b	Present results of all statistical syntheses conducted. If meta-analysis was done, present for each the summary estimate and its precision (e.g. confidence/credible interval) and measures of statistical heterogeneity. If comparing groups, describe the direction of the effect.	NA
	20c	Present results of all investigations of possible causes of heterogeneity among study results.	NA
	20d	Present results of all sensitivity analyses conducted to assess the robustness of the synthesized results.	NA
Reporting biases	21	Present assessments of risk of bias due to missing results (arising from reporting biases) for each synthesis assessed.	NA
Certainty of evidence	22	Present assessments of certainty (or confidence) in the body of evidence for each outcome assessed.	NA
**DISCUSSION**			
Discussion	23a	Provide a general interpretation of the results in the context of other evidence.	12
	23b	Discuss any limitations of the evidence included in the review.	13
	23c	Discuss any limitations of the review processes used.	13-14
	23d	Discuss implications of the results for practice, policy, and future research.	14-15
**OTHER INFORMATION**			
Registration and protocol	24a	Provide registration information for the review, including register name and registration number, or state that the review was not registered.	6
	24b	Indicate where the review protocol can be accessed, or state that a protocol was not prepared.	6
	24c	Describe and explain any amendments to information provided at registration or in the protocol.	6
Support	25	Describe sources of financial or non-financial support for the review, and the role of the funders or sponsors in the review.	1
Competing interests	26	Declare any competing interests of review authors.	2

For peer review only - http://bmjopen.bmj.com/site/about/guidelines.xhtml

PRISMA 2020 Checklist

Section and Topic	Item #	Checklist item	Location where item is reported
Availability of data, code and other materials	27	Report which of the following are publicly available and where they can be found: template data collection forms; data extracted from included studies; data used for all analyses; analytic code; any other materials used in the review.	2

From: Page MJ, McKenzie JE, Bossuyt PM, Boutron I, Hoffmann TC, Mulrow CD, et al. The PRISMA 2020 statement: an updated guideline for reporting systematic reviews. BMJ 2021;372:n71. doi: 10.1136/bmj.n71

For more information, visit: http://www.prisma-statement.org/

For peer review only
